# The mosquitoes (Diptera: Culicidae) of Tunisia: updated checklist and new distribution data

**DOI:** 10.1051/parasite/2026018

**Published:** 2026-04-14

**Authors:** Ahmed Ouni, Adel Rhim, Youmna M’Ghirbi, Francis Schaffner, Ali Bouattour

**Affiliations:** 1 Laboratoire des Virus, Vecteurs et Hôtes (LR20IPT02), Institut Pasteur de Tunis, Université Tunis El Manar Tunis 1002 Tunisia; 2 BioSys – EI Schaffner Francis Steinbach France

**Keywords:** Checklist, Culicidae, Tunisia, North Africa, Distribution, Taxonomy, Vector surveillance

## Abstract

In the context of climate change and rapid environmental changes in the Mediterranean basin, accurate and up-to-date mosquito distribution data are essential to support the control of mosquito-borne diseases, which remain a major public health concern. Here, we report the results of intermittent, nationwide, cross-sectional mosquito sampling missions conducted in Tunisia between 2013 and 2023 to update the Culicidae checklist and document current species distribution of species. A total of 35 species were collected, compared with 49 species previously reported in the Tunisian literature. The primary malaria vectors *Anopheles labranchiae* and *An. claviger sensu stricto* were still recorded in the north, while *An. multicolor* and *An. sergentii* were found in the south, indicating that vigilance remains necessary following malaria elimination. The most abundant and widespread species were *Aedes caspius*, *Ae. detritus*, *Culex perexiguus* and *Cx. pipiens.* The latter two are recognised as vectors of pathogens such as West Nile virus and therefore require sustained surveillance. Several mosquito species previously reported in the literature were not detected during our surveys. The invasive species *Ae. Albopictus*, recently established in Tunisia, requires particular monitoring due to its spread to several regions of the country.

## Abstract (fr)

Dans un contexte de changement climatique et de modifications environnementales rapides dans le bassin méditerranéen, des données précises et actualisées sur la distribution des moustiques sont essentielles pour lutter contre les maladies transmises par ces insectes, qui constituent un problème majeur de santé publique. Nous présentons ici les résultats de missions d’échantillonnage transversales, menées de manière intermittente et à l’échelle nationale en Tunisie entre 2013 et 2023, afin de mettre à jour la liste des Culicidae et de documenter la distribution actuelle des espèces. Au total, 35 espèces ont été recensées, contre 49 précédemment répertoriées dans la littérature tunisienne. Les principaux vecteurs du paludisme, *Anopheles labranchiae* et *An. claviger sensu stricto*, ont été observés dans le nord du pays, tandis qu’*An. multicolor* et *An. sergentii* ont été trouvés dans le sud, ce qui indique que la vigilance reste nécessaire après l’élimination du paludisme. Les espèces les plus abondantes et les plus répandues étaient *Aedes caspius*, *Ae. detritus*, *Culex perexiguus* et *Cx. pipiens*. Ces deux dernières espèces sont reconnues comme vecteurs de pathogènes tels que le virus du Nil occidental et nécessitent donc une surveillance continue. Plusieurs espèces de moustiques précédemment décrites dans la littérature n’ont pas été détectées lors de nos enquêtes. L’espèce invasive *Aedes albopictus*, récemment implantée en Tunisie, requiert une surveillance particulière en raison de sa propagation dans plusieurs régions du pays.

## Introduction

The family Culicidae, known as mosquitoes, of the Diptera order and Nematocera suborder, comprises about 3,583 described species that are globally distributed [57]. Only a small fraction of mosquito species play a role in transmitting pathogens to humans and animals. According to the World Health Organization (WHO), mosquito-borne diseases account for about 17% of all infectious diseases [168]. Mosquito species of the genera *Aedes* and *Culex* play a principal role in the transmission of several viruses and Filariae. *Anopheles* mosquitoes transmit malaria parasites and are responsible for the most serious mosquito-borne disease globally; they cause thousands of deaths, primarily in Africa [169].

In Tunisia, little is known about mosquito fauna. Records of mosquito species collected in the country are found in the few papers published around the mid-20th century [25, 64, 101, 120]. Additional studies are reported in more recent publications [7, 18, 70, 144]. The invasive species *Aedes albopictus* was reported in Tunisia subsequently [8, 12]. The literature cites 49 mosquito species belonging to 6 genera (*Aedes*, *Anopheles*, *Culex*, *Culiseta*, *Orthopodomyia* and *Uranotaenia*) [12, 17, 108]. Meanwhile the classification of the tribe Aedini underwent a major taxonomic change in 2000 [98] and in several subsequent articles [99, 100, 165], leading to some confusion regarding the identification and use of species names. Moreover, some species may disappear and others emerge as a result of climate change, environmental modifications, demographic growth and urbanisation, which have a significant impact on water bodies, including wastewater.

Earlier publications were particularly interested in the Tunisian Culicidae fauna solely because of the role played by *Anopheles* in the transmission of malaria parasites [24]. In the early 20th century, several epidemics of malaria, which was endemic in the country, were caused by *Plasmodium falciparum* [4, 24]. Malaria was officially eradicated in 1979 thanks to the national malaria eradication programme launched in Tunisia in 1966 with the support of WHO [24, 25]. Its eradication lowered interest in mosquito and mosquito-borne disease studies in Tunisia.

Today, its role as an important transit country in North Africa for human travel, livestock trade and migratory birds puts Tunisia at high risk for the (re-) emergence of arboviruses. Importantly, as part of the geographical unit separating sub-Saharan Africa from Mediterranean Europe, Tunisia has a range of environmental zones that can support rich diversity and the occurence of potential mosquito vectors capable of transmitting several pathogens to animals and humans. The precise identification of mosquito species and knowledge of their distributions and bio-ecology is therefore important to support the surveillance and control of mosquito-borne diseases [26, 57].

To update the mosquito fauna and their distribution, we report the results of several cross-sectional mosquito field studies across the country during 2013–2023. Carried out from the north to the south of the country, these surveys sought to investigate as many mosquito habitats as possible.

## Materials and methods

### Study area

**Figure 1:**
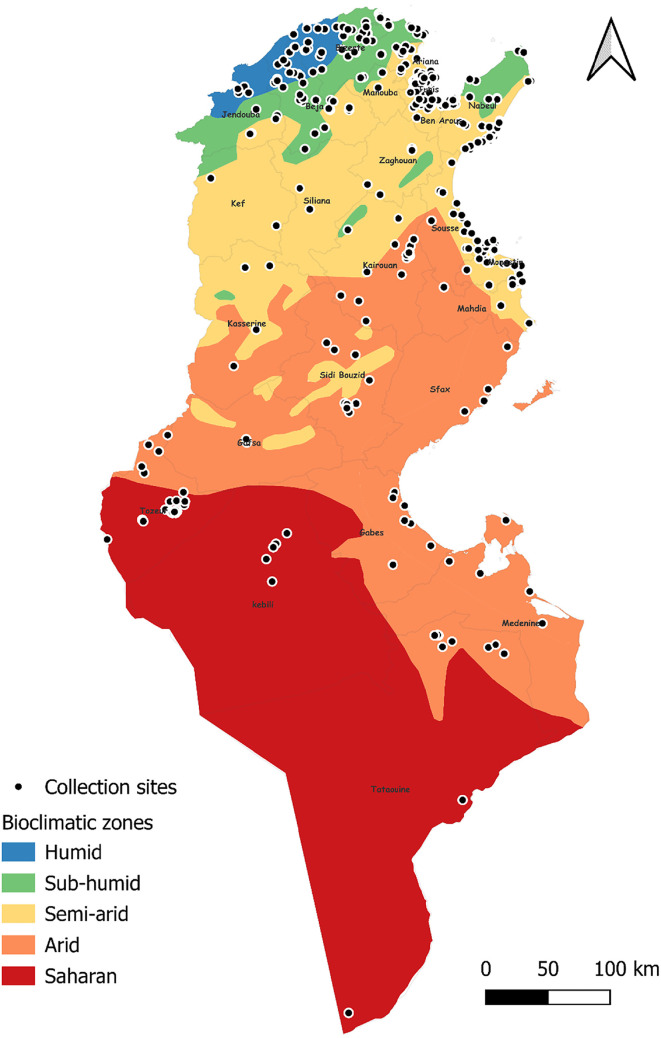
Bioclimatic map of Tunisia showing mosquito sampling locations (2013–2023), produced using QGIS Software (Lima 3.32.3).

Tunisia is a North African country (33°58′48″ N; 9°32′24″ E) bordered by the Mediterranean Sea to the north and east, Algeria to the west, and Libya to the south-east. The country is mountainous in the northwest; the eastern Mediterranean coast is a large plain, whereas the south is hot and dry, progressively merging into the Sahara. Based on the bioclimatic classification of Emberger (1960) [43] and Gounot’s (1995) [50], Tunisia can be divided from north to south into five main bioclimatic zones (Fig. 1): (i) humid (annual rainfall ranging from 800 to 1 200 mm/year), subhumid (600–800 mm/year), semi-arid (300–600 mm/year), arid (100–300 mm/year) and Saharan or desert zones (up to/less than 100 mm/year).

### Mosquito collection and identification

Mosquito collection was carried out over 10 years (2013–2023). Several cross-sectional investigations of randomly selected potential breeding sites (stagnant water) were conducted in the five bioclimatic zones of the country and focused mainly on aquatic immature stages of mosquitoes (larvae and pupae). These surveys were conducted across different regions during several field missions, with variable sampling intensity (Fig. 1). Then, following the West Nile fever outbreak in Tunisia in 2012, field missions were intensified to identify the mosquito vectors involved. In 2020, field activities were reduced due to the COVID-19 pandemic, resulting in fewer surveys. An additional cross-sectional survey along a north-south transect was carried out in 2017 as part of the VectorNet project (contracted by the European Centre for Disease Prevention and Control [ECDC] and the European Food Safety Authority [EFSA]) which supports the collection of distribution data on vectors of pathogens related to both human and animal health. Sampling sites were selected to cover the main Tunisian bioclimatic zones and the majority of larval habitats (Fig. 1), whether natural (wadis, sebkhas, ponds, streams, swamps, tree holes, *etc.*) or artificial/man-made (tyres, channel, puddles, *etc.*). These were chosen based on the national hydrological map and on the information provided by the environmental health officers (Ministry of Health) of each locality. Humid regions receive high levels of rainfall and consequently have more stagnant water, which provides potential breeding sites; therefore, these regions were surveyed more frequently. A subset of key sites was revisited over multiple years to confirm the presence of mosquito species.

We applied a standardised procedure for sampling mosquito larvae in stagnant water, as described in WHO technical manuals [167] and commonly used in entomology, public health, and vector surveillance. Using a standard long-handled 500 mL dipper, mosquito immatures were collected from different breeding sites (natural and human-made water collections), including wadi banks, swamps, pools, drainage sites, catch basins and ditches. The standard dipper was immersed approximately ten times at the edge of large breeding sites. For small habitats, such as tree holes and water vessels, we used a 50 mL pipette with a rubber bulb to aspirate the water. Only 3rd or 4th larvae stages were preserved in ethanol (70%) for morphological identification. First and second stage larvae and pupae were kept alive until obtaining 4th larval stages or adults for accurate species identification.

In addition to collecting immature stages, we trapped adult mosquitoes during the warm season (May–November) using two types of traps: (i) CDC miniature light traps (John W. Hock Co., Gainesville, FL, USA) with dry ice as a source of CO_2,_ and (ii) BG-Sentinel and BG-Lure traps (Biogents, Regensburg, Germany). CDC light traps were installed near animal shelters, dwellings and larval habitats and generally operated from approximately 6 pm to 6 am to cover nocturnal activity. BG-Sentinel traps were placed close to human habitations to collect adult mosquitoes active during the day, mainly *Ae. albopictus*, usually between 7 am and 5 pm. Depending on mission objectives and site accessibility, up to eight CDC light traps per site per night and up to three BG-Sentinel traps per site per day were deployed for one to three consecutive nights, with placement adapted to local conditions (near dwellings, animal shelters and/or productive larval habitats).

The captured larvae and adults were observed under a binocular microscope. Specimens were identified to the species level using standard dichotomic keys [123, 124] and a computer-aided key [18]. Field observations were considered for biting behaviour of adults. While biting, mosquitoes were caught in tubes (crackers) and kept alive for identification.

### Larval habitats characterisation

During sampling, breeding habitats were classified as natural or artificial and characterised according to the depth and the vegetation, and geographic coordinates were recorded using a GPS (Garmin-eTrex 30). Physicochemical parameters (salinity, pH and temperature) were measured *in situ* using portable meters (Starter Pen Meters ST20S and ST10; OHAUS, Parsippany, NJ, USA) when possible. However, measurements were not systematically performed at all breeding sites due to logistical constraints and site accessibility.

## Results and discussion

### Characterisation of larval breeding sites

We collected and identified approximately 30,000 mosquito specimens during these decade-long successive field studies throughout the Tunisia’s five bioclimatic zones. Specimens included around 26,300 immature stages collected from breeding sites and 3,700 adults caught by CDC and BG-Sentinel traps and those obtained from pupae. Mosquito larvae were found in a variety of water collections. Among the 2,425 water bodies surveyed for the presence of mosquito larvae, 1,800 were found to be positive for mosquito larvae. Negative stagnant waters were often highly eutrophic (polluted, covered by duckweed, *etc.*), contaminated by industrial effluents in some wadis, or colonised by *Gambusia* spp. (introduced in the past for the control of mosquito larvae). Some larval breeding sites, mainly in urban areas, were treated with insecticides by health authorities.

Our surveys highlight a variety of mosquito species occupying diverse larval habitats. Mosquito breeding sites differed from one bioclimatic zone to another. Wadis and tree holes were the most common natural breeding sites in the north, while in the center and the south, sabkhas were the main habitat. In urban environments, open-air canals (which frequently contain greywater) often serve as the primary larval breeding grounds. The prospected larval habitats included artificial (canals, pools) and natural (wadi, sabkhas, swamps) sites with temporary (pools, unused tyres), permanent (sabkha) or semi-permanent (seasonal ponds), depending on their stability (see Supplementary Table 1 for details).

In the investigated habitats, 12 of all identified mosquito species were found in all bioclimatic zones and thus considered to be common, such as *Cx. pipiens* (see Supplementary Table 1 for details). These species exhibit a wide geographical distribution, indicating their adaptability to diverse environmental conditions. Conversely, other mosquito species were restricted to specific bioclimatic zones such as *An. plumbeus* which occurs exclusively in the humid northern regions, while other species such as *An. sergentii* were found exclusively in the southern region where they were collected mainly in the watercourses of the oases (see Supplementary Table 1 for details). Specific habitats such as tree holes and rock pools were occupied by specialised mosquito species.

In the investigated habitats, water temperature ranged from 15 to 35 °C, salinity from 0.1 to 66.2 g/L (NaCl), and pH from 7.08 to 10.8, reflecting a gradient from freshwaters to hypersaline sebkhas and salt marshes. Depths ranged from 5 cm to 2 m.

The vegetation in mosquito breeding sites varied widely. In wadis and hill lakes, the Characeae and *Potamogeton* were the dominant vegetation, submerged, while salt marshes and sabkhas were covered with halophyte plants, *such as Salicornia*. Sabkhas subjected to flooding with rainwater were covered by *Phragmites*, *Juncus* and *Typha*.

### Mosquito checklist

During our surveys we collected and identified around 30,000 mosquito specimens from 35 species belonging to 6 genera: *Anopheles* (10 species), *Aedes* (12 species), *Culex* (9 species), *Culiseta* (2 species), *Orthopodomyia* (1 species) and *Uranotaenia* (1 species) (see Supplementary Table 2 for details). The *Culex* genus was most represented in our collection (66%), followed by *Aedes* (17%), *Culiseta* (9%), *Anopheles* (6%) and *Uranotaenia* (2%), and only a few specimens of *Orthopodomyia.*

#### Species identified during the current investigation

##### (See Supplementary Tables 1 and 2 for details) Subfamily Anophelinae Grassi, 1900 Genus *Anopheles* Meigen, 1818

Of the 13 *Anopheles* (*An*.) species mentioned in the literature, only 10 were identified during our survey: *An. algeriensis*, *An. cinereus*, *An. claviger*, *An. labranchiae*, *An. marteri*, *An. multicolor*, *An. petragnani*, *An. plumbeus*, *An. sergentii* and *An. ziemanni.* These species were all reported in neighbouring Algeria [146] and Morocco [149]. While *Anopheles coustani*, *An. dthali* and *An. superpictus* have been reported in Tunisia, we did not find them during our surveys.

###### *Anopheles* (*Anopheles*) *algeriensis* Theobald, 1903

**Figure S1.**
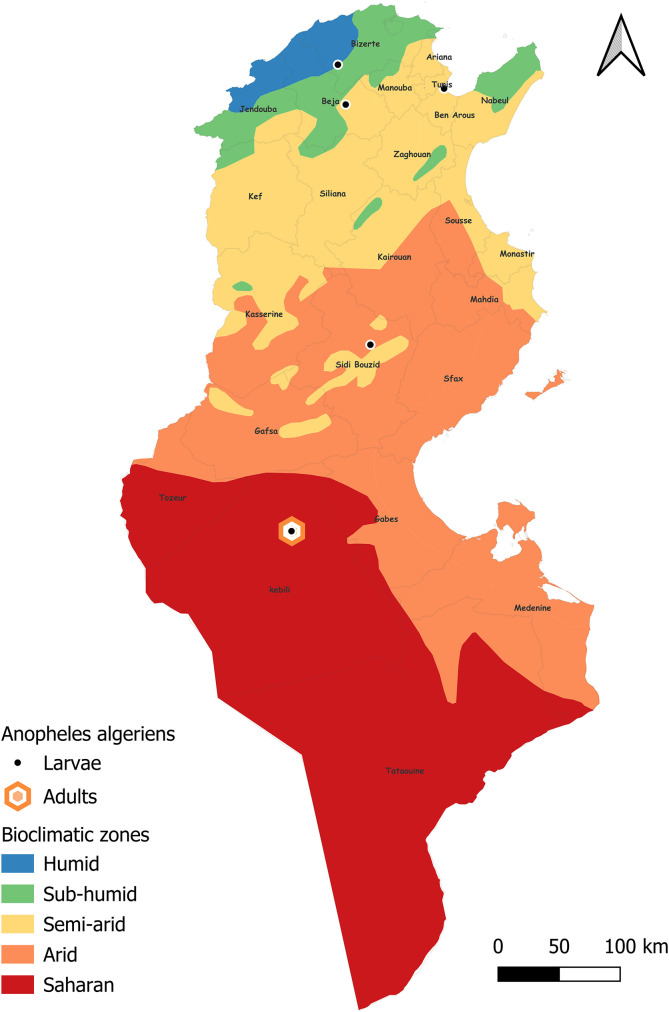
Geographical distribution of *Anopheles algeriensis* in Tunisia (2013–2023) across the different bioclimatic zones.

In both humid and subhumid zones, we collected *An. algeriensis* alongside *An. labranchiae*; in arid areas, it was associated with *An. sergentii* (Fig. S1). We found larvae in swamps, slow-moving streams, marshes, wadi banks, ponds and hill lakes overgrown with vegetation. The breeding sites were typically shaded and the water in them was consistently fresh, although it was occasionally saline.

The species was observed in Algeria [52], Morocco [72, 150] and Libya [17] as well. It is also present in Europe and elsewhere, including Afghanistan [59]. The information on the biting behaviour of *An. algeriensis* adults is contradictory, probably because of the rarity of observations. However, during field visits at dusk in Kebili (southern region), intense host-seeking activity of *An. algeriensis* was observed in the immediate vicinity of breeding sites.

###### *Anopheles* (*Cellia*) *cinereus* Theobald, 1901

**Figure S2.**
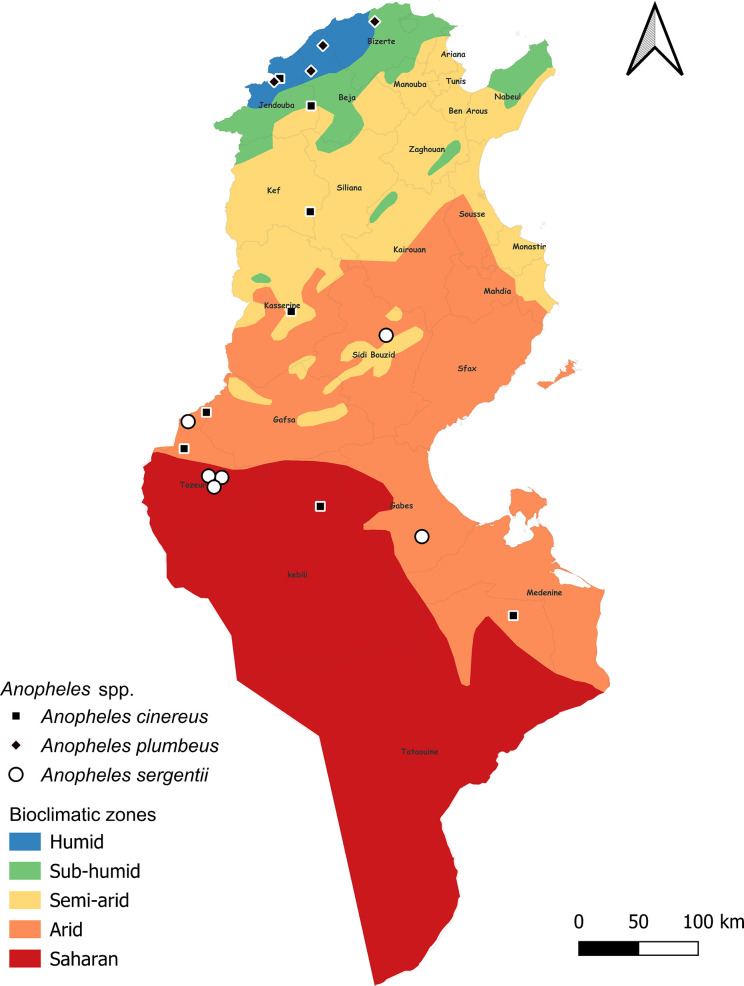
Geographical distribution of *Anopheles* spp. [*Anopheles algeriensis*, *Anopheles cinereus*, *Anopheles multicolor*, *Anopheles plumbeus* and *Anopheles sergentii*] in Tunisia (2013–2023) across the different bioclimatic zones.

The results of our survey indicate that *An. cinereus* is present in all bioclimatic zones of Tunisia (Fig. S2). The species has a wide distribution, and immatures were found in wadi banks, swamps, ponds, pools, drainages and catch basins, and can be collected on the surface of Spirogyra mats. It is typically found in association with *An*. *labranchiae* in the north and *An*. *sergentii* in the south. Previously identified as *An. hispaniola*, this species was cited as *Anopheles* (*Cellia*) *cinereus hispaniola* by Theobald 1903 [18]. Several authors have reported the presence of *An*. *cinereus* in Algeria and in Morocco [9, 152].

###### *Anopheles* (*Anopheles*) *claviger* (Meigen, 1804)

*Anopheles claviger sensu stricto* was identified exclusively in the Tunisia’s northwest humid zone, where the cool climate is typical of oak forests [36°52′0.85″ N; 8°42′51.66″ E]; it is considered to be rare in Tunisia. Larvae were collected with very low density from ponds that were densely vegetated with aquatic plants. *Anopheles claviger* is also found in mountainous regions of the Maghreb, including Morocco and Algeria, but its presence in Libya requires confirmation [108, 123, 152]. According to Robert *et al*. (2019) [108], *An. claviger* is present from Afghanistan to England and in the northernmost areas of Europe, including Sweden and Norway. Although it was considered to be a malaria vector in the Middle East, its vectorial role is considered to be neglected in North Africa due to its rarity [88]. *Anopheles claviger sensu stricto* is morphologically similar to its sibling species *An. petragnani*, making differentiation difficult. Some earlier records of *An. claviger* might therefore belong to *An. petragnani*.

###### *Anopheles* (*Anopheles*) *labranchiae* Falleroni, 1926

**Figure S3.**
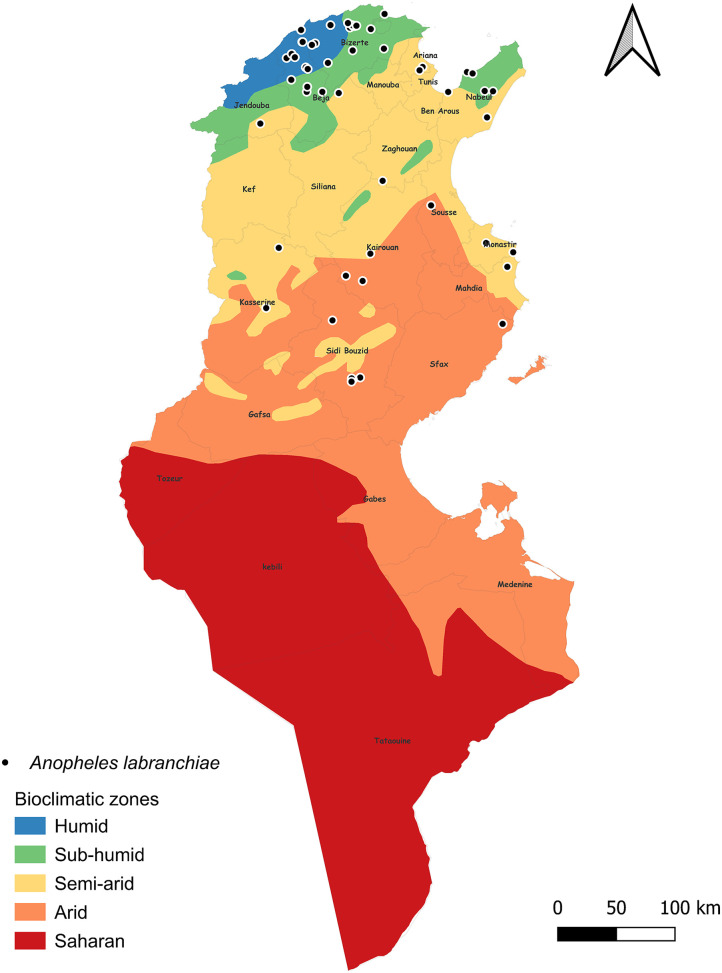
Geographical distribution of *Anopheles labranchiae* in Tunisia (2013–2023) across the different bioclimatic zones.

*Anopheles labranchiae* is the only species in Tunisia of the Maculipennis Complex. *Anopheles labranchiae* can be found in areas up to 1,900 m in elevation, with a distribution extending from coastal areas to the Sahara in western Palaearctic regions. During our survey, we collected this species in several habitats (wadis, hill lakes, swamps, ponds and abandoned quarries) from the north of the country towards the center, up to Sidi Bouzid (in Oued Leben) (Fig. S3). The species is frequently encountered in humid, sub-humid, and semi-arid areas, although it is rare in arid regions. The larvae of this mosquito prefer stagnant, fresh, unpolluted waters exposed to sunlight, with vegetation dominated by *Ranunculus* and *Ceratophyllum*. The submerged vegetation typical of its habitats is mainly composed of *Potamogeton* and members of the Characeae. The habitats are often bordered by tamarisk (*Tamarix* spp.) trees. The current distribution of this species suggests that this mosquito has disappeared from several areas, probably due to environmental changes leading to the loss of suitable habitats. Indeed, this species, sometimes reported as *An*. *maculipennis*, was also found in coastal areas where urbanisation has modified the environment. During our field investigations, biting of humans was observed, notably in northern areas near low-water wadis or hill lakes.

*Anopheles labranchiae* was considered to be a major malaria vector in the north of Tunisia [13, 14, 25]. Several studies have confirmed that this species can transmit *Plasmodium falciparum* [110, 164]. The regions where this species is present can therefore be considered high-risk sites for the re-emergence of malaria [2].

###### *Anopheles* (*Anopheles*) *marteri* Sénévet & Prunnelle, 1927

*Anopheles marteri* is a zoophilic species that we observed in the forested regions of northwest Tunisia, frequently associated with *An. petragnani*. Its larvae were typically found in freshwater springs and streams in Sidi Mechreg [37°9′36.35″ N; 9°7′1.45″ E]. *Anopheles marteri* is considered to be rare in Tunisia and given its strong preference for animal hosts, it does not play a role in the transmission of human disease pathogens [32]. First described in Algeria, this species is also present in several Mediterranean countries, particularly in the mountainous areas of North Africa [48, 63, 118].

###### *Anopheles* (*Cellia*) *multicolor* Cambouliu, 1902

**Figure S4.**
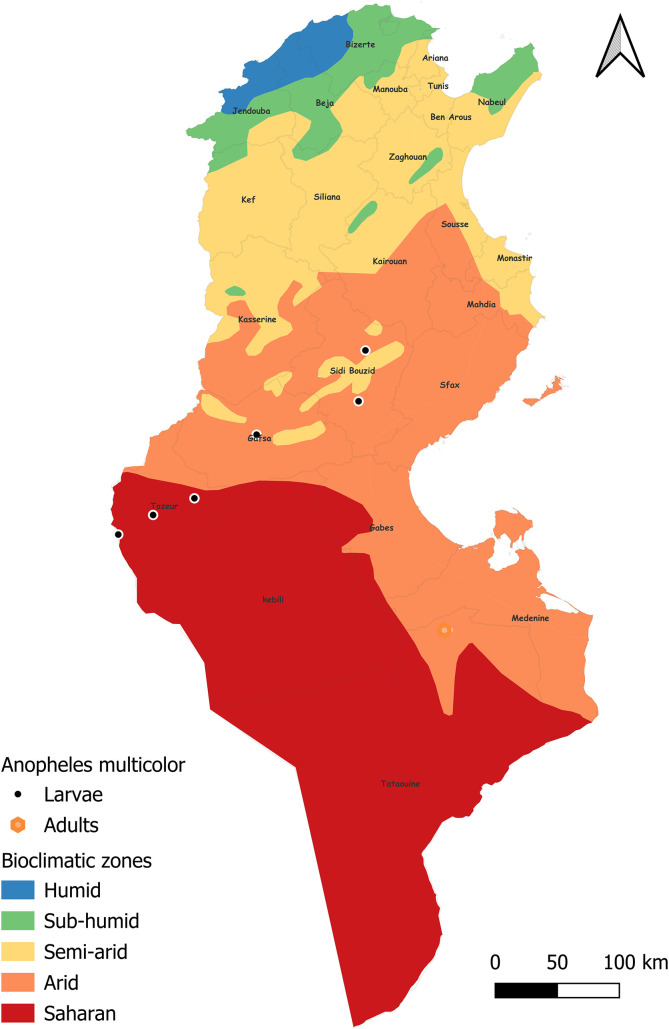
Geographical distribution of *Anopheles multicolor* in Tunisia (2013–2023) across the different bioclimatic zones.

*Anopheles multicolor* is typically a halophilic species that we rarely observed outside hyper-salty environments in Tunisia. We collected the larvae mostly in the brackish waters of arid, Saharan areas and around oases, where it is associated with *An. sergentii* (Fig. S4). The species tolerates high salt concentrations but is never found in puddles near the sea and sometimes breeds in organically rich water. It is also typically found in arid and semi-arid areas throughout North Africa from Morocco to Egypt, and as far east as Pakistan [57]. Adults are abundant at the end of summer and the beginning of autumn and have a wide range of dispersion. Females are mainly exophilic and zoophagic, but they also bite humans. The species was incriminated in the transmission of malaria in the south of Tunisia [25] and also suspected as a vector of *P. falciparum* in Egypt [42, 65].

###### *Anopheles* (*Anopheles*) *petragnani* Del Vecchio, 1939

During our field investigations in northern Tunisia, we found *An. petragnani* specifically in the oak forests [36°47′59.87″ N; 8°40′46.22″ E]. This species exhibits a marked preference for bodies of cool, clear water with abundant floating vegetation, often cohabiting with other forest-dwelling mosquito species. Previous reports documented *An. petragnani* in coastal regions [18, 84]. Its presence in the north suggests that its suitable habitats are restricted to the interior mountainous areas of Tunisia. Reports from northeastern Algeria (Souk Ahras) support the affinity of this mosquito for similar mountainous zones [52].

###### *Anopheles (Anopheles) plumbeus* Stephens, 1828

*Anopheles plumbeus* is a rare species that we found in the forests of northwestern Tunisia. Its larvae breed in water that accumulates in the holes of cork oak trees (*Quercus suber*) (Fig. S2). This species was reported in Algeria [119] and is abundant throughout Europe and can be found as far east as Iran [112]. During our prospections, numbers of aggressive females were observed near larval breeding sites. The species is known to be aggressive towards humans, mammals, birds and reptiles [19]. Some authors suggest that *An. plumbeus* can act as a vector of *Plasmodium falciparum* [113, 145]. Experimentally, it can transmit Plasmodiidae, Flaviviridae and Filariae [79, 81, 113].

###### *Anopheles* (*Cellia*) *sergentii* (Theobald, 1907)

During our surveys, *Anopheles sergentii*, an anthropophilic species, was identified in clear fresh or slightly brackish waters, including wadi beds and oasis stream channels, along with other species of the genus *Anopheles* (Fig. S2). Although historical records attest the presence of this species in semi-arid and arid areas of Tunisia, our results suggest a reduction in its habitat towards arid and pre-Saharan zones. In North Africa, *An. sergentii* was listed in Libya [66], Algeria [53] and Morocco [151]. It has a wide distribution from Africa to Asia [138] (Sinka *et al*., 2010) especially in arid zones. This mosquito is considered to be the main vector of malaria in southern Tunisia [25, 144]. Its vector role is also confirmed in Morocco [45], Algeria [97] and Egypt [78].

###### *Anopheles (Anopheles) ziemanni* Grünberg, 1902

*Anopheles ziemanni* is a rare species that we collected in northern Tunisia, where it coexists with *An. labranchiae*. *Anopheles ziemanni* larvae typically develop in large, vegetation-littered wet fields like marshes and the grassy edges of large ponds [36°57′ N; 9°0′ E]. Initially classified as a subspecies of *An. coustani* due to the indistinguishable larvae, it was subsequently elevated to full species status [63]. This change explains why older records may incorrectly refer to *An. coustani* in the Maghreb, where it is present only in Algeria [122]. The presence of *An. ziemanni* in Libya needs confirmation [96, 108]. In North Africa, *An. ziemanni* does not appear to play a significant role in the transmission of human pathogens, although it may serve as a vector for Filariae in animals [67].

##### Subfamily Culicinae Meigen, 1818 Genus *Aedes* Meigen, 1818 [Tribe Aedini Neveu-Lemaire, 1902]

Sixteen species of the genus *Aedes* were cited in Tunisia. Of these, only 12 were identified during the present survey: *Ae. albopictus*, *Ae. berlandi*, *Ae. caspius*, *Ae. coluzzii*, *Ae. detritus*, *Ae. dorsalis*, *Ae. echinus*, *Ae. geniculatus*, *Ae. mariae*, *Ae. pulcritarsis*, *Ae. vexans* and *Ae. vittatus*. Among the *Aedes* species mentioned in the literature, we did not find *Ae. aegypti*, *Ae. albineus*, *Ae. cinereus* or *Ae. zammiti*.

###### *Aedes* (*Stegomyia*) *albopictus* (Skuse, 1894)

**Figure S5.**
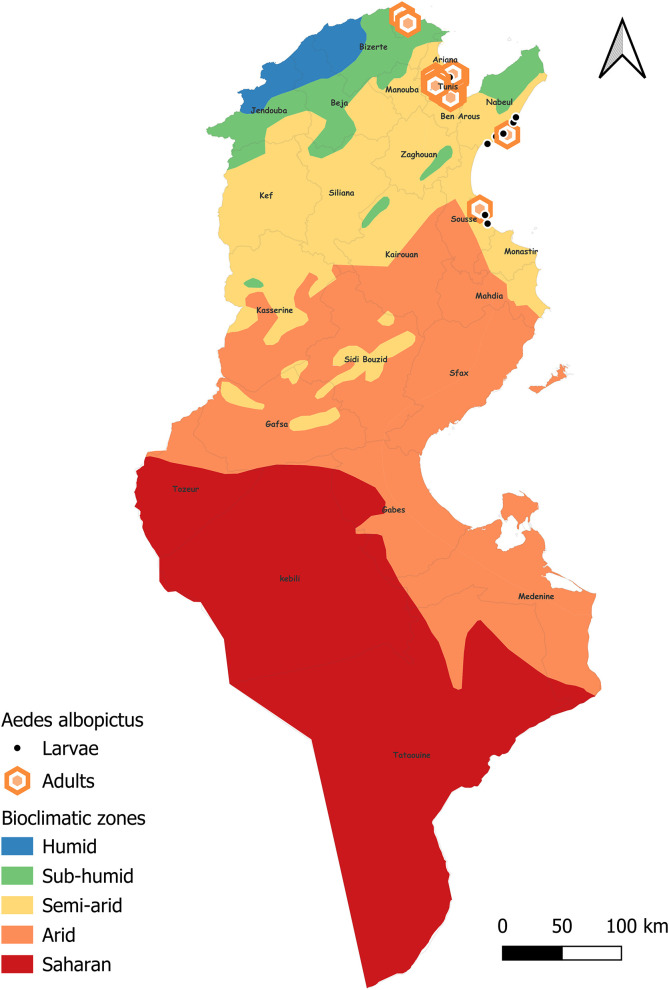
Geographical distribution of *Aedes albopictus* in Tunisia (2013–2023), by life stage (larvae and adults) across the different bioclimatic zones.

*Aedes albopictus* (Asian tiger mosquito) was recently identified for the first time in Tunisia [12] around Carthage, a city 20 km north of the Tunisian capital, Tunis. Following this initial discovery, the distribution area of the species proved to encompass several districts of Tunis. We subsequently noted its presence in the northern region of Bizerte (60 km northwest of Tunis) and later in the southern region of Sousse (120 km south of Tunis) in 2023 (Fig. S5**)**.

This species has been collected from urban areas, particularly gardens, cemeteries and rubbish tips. Known for its resilience and adaptability, *Ae. albopictus* colonises artificial habitats such as used tyres and small water containers with fresh water.

It is a proven vector of several arboviruses, including chikungunya, dengue and Zika viruses, and has been experimentally proven to be capable of transmitting many other arboviruses, including Japanese encephalitis, Rift Valley fever and West Nile viruses [11, 68, 81, 95, 140, 143]. In Tunisia, it has not yet been implicated in disease transmission. The rapid spread of *Ae. albopictus* in the Americas, Europe and Africa in recent decades highlights its invasive potential [80]*.*

###### *Aedes* (*Ochlerotatus*) *berlandi* Séguy, 1921

**Figure S6.**
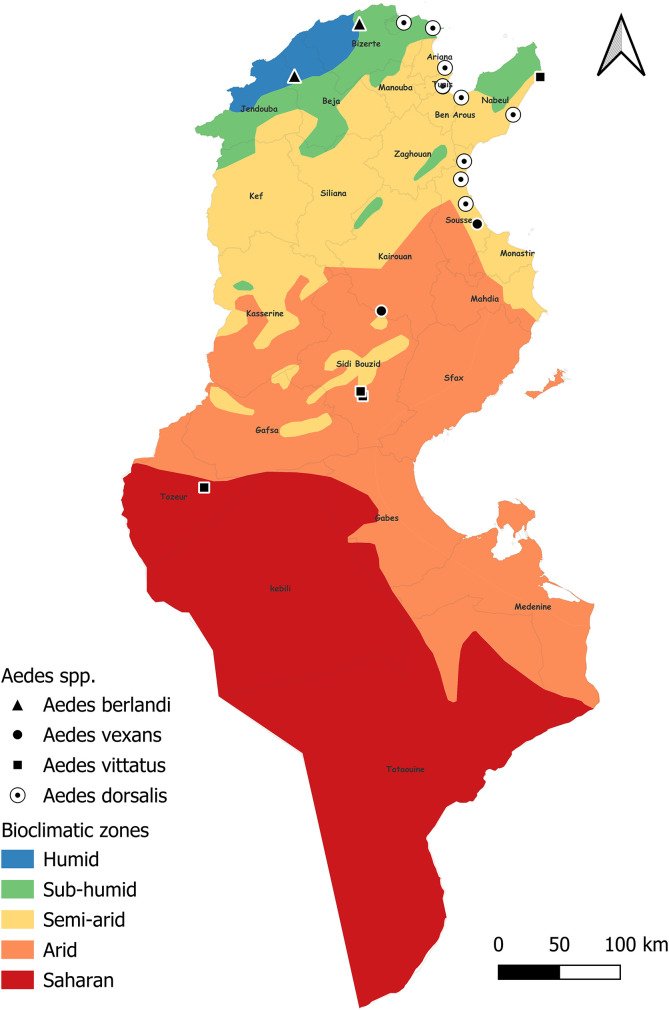
Geographical distribution of selected *Aedes* spp. [*Aedes berlandi*, *Aedes dorsalis, Aedes vexans* and *Aedes vitatus*] recorded in Tunisia (2013–2023) across the different bioclimatic zones.

*Aedes berlandi* is a rare species of phytotelma mosquito found in association with *Ae. echinus* and *An. plumbeus* in northern Tunisia’s humid, forested regions. Its larvae were collected from the water-filled cavities, predominantly of oak trees (Fig. S6); the water is typically alkaline and rich in organic matter and tannins. The distribution of *Ae. berlandi* is concentrated primarily in the western Mediterranean region, with additional reports from Algeria, France, Italy, Morocco, Portugal and Spain [86, 106, 108].

###### *Aedes* (*Ochlerotatus*) *caspius* (Pallas, 1771)

**Figure S7.**
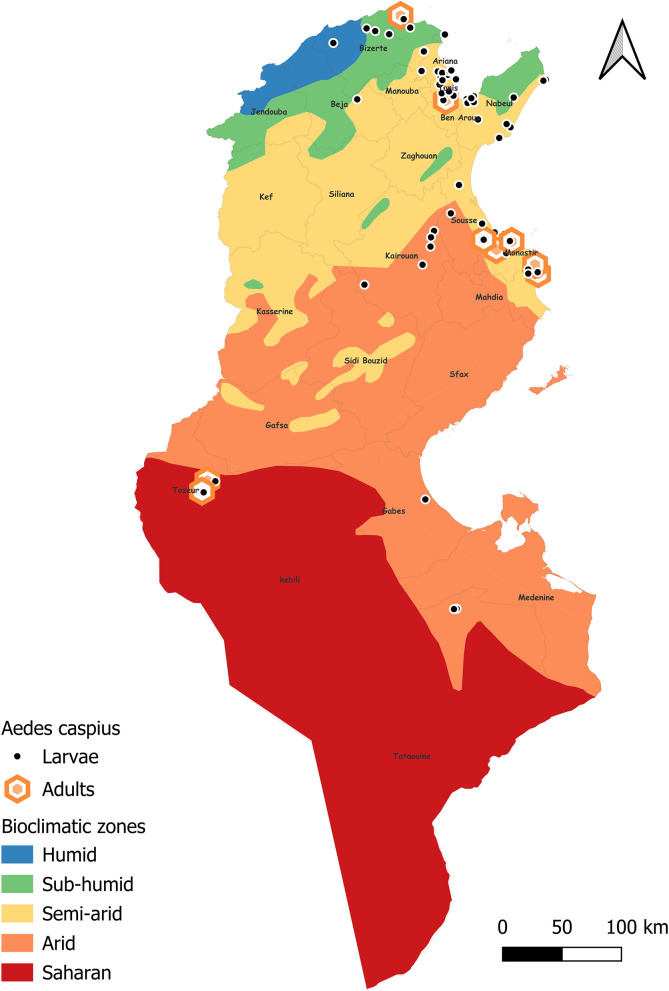
Geographical distribution of *Aedes caspius* in Tunisia (2013–2023), by life stage (larvae and adults) across the different bioclimatic zones.

*Aedes caspius* is one of the most widespread and abundant mosquito species in Tunisia. It was collected during this study, usually at high densities, in all bioclimatic zones (Fig. S7). This halophilic species is commonly found in coastal areas in sabkhas, salt marshes and ponds formed by rainwater or water discharged from food factories, as well as in artificial habitats such as pools and ditches. It is considered to be highly adaptable to saline and brackish water environments, one factor that has contributed to its wide distribution. This mosquito was found in water bodies ranging from freshwater to brackish environments, such as coastal sebkhas (1–59.5 g/L NaCl; pH ~7–8.9), illustrating its broad salinity tolerance.

A comparison of earlier data with our monitoring findings showed a reduction in the number of larval habitats of this species due to increasing urbanisation, particularly in coastal areas. The species has been extensively documented in North Africa, with records extending from Algeria [119], Morocco [48] and Libya [108]. Globally, the species is distributed throughout Europe and the Middle East. From a public health perspective, *Ae. caspius* is a significant vector for various viruses, including Rift Valley fever virus, and has been proposed as a vector for *Francisella tularensis* [37, 38, 61].

###### *Aedes* (*Ochlerotatus*) *coluzzii* Rioux, Guilvard & Pasteur, 1998

*Aedes coluzzii* is closely related to *Ae. detritus*, from which it cannot be distinguished morphologically [104] (Rioux *et al*., 1998). We recently confirmed its presence in Tunisia using primers based on ITS2 rDNA sequences (data in preparation), based on specimens collected from sebkhat Korba (northeastern Tunisia) [36°35′2.04″ N; 10°52′10.52″ E], associated with *Ae. detritus*. This confirms the observations of [104] Rioux *et al*. (1998) of this species in certain sites in Tunisia. It is in fact genetically isolated and shows a preference for hyperhaline environments, making it particularly well adapted to arid zones. *Aedes coluzzii* has also been reported in Algeria [73] and Morocco [151].

###### *Aedes* (*Ochlerotatus*) *detritus* (Haliday, 1833)

**Figure S8.**
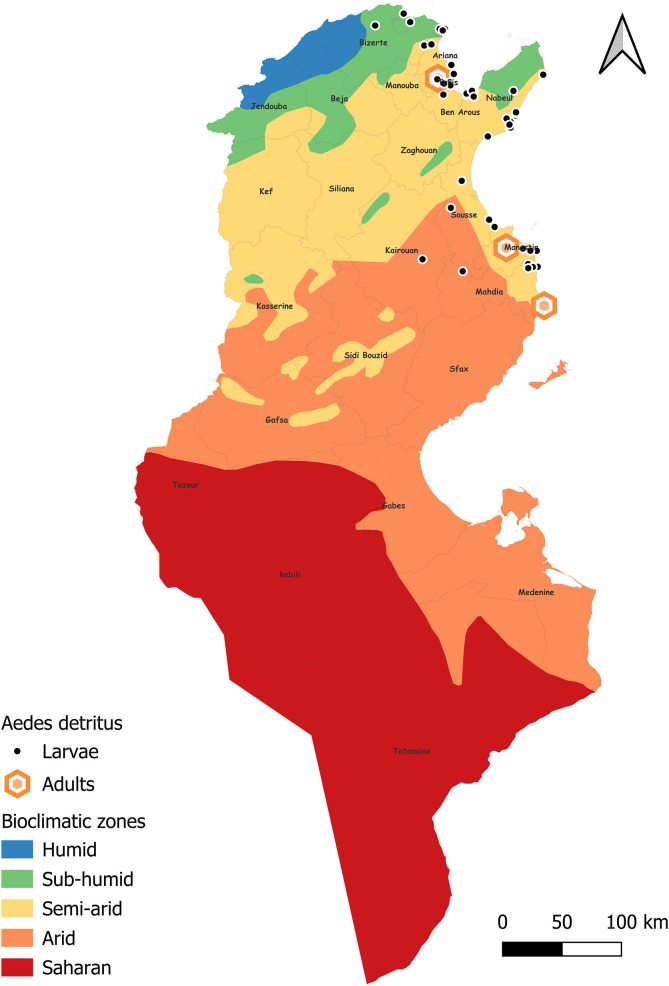
Geographical distribution of *Aedes detritus* in Tunisia (2013–2023), by life stage (larvae and adults) across the different bioclimatic zones.

*Aedes detritus* is a halophilic species that is frequent and abundant in Tunisia and often develops in the same habitat as *Ae*. *caspius*. We collected it in all bioclimatic zones in Tunisia, where it is most commonly found in sabkhas and coastal salt marshes (Fig. S8). It was also observed to breed in habitats such as wadi banks and swamps, and in artificial water sources such as pools, unused wells, drainages, catch basins, and ditches, with higher population densities in stagnant water with high salinity, particularly along coastal and arid zones. Our physicochemical data showed that *Ae. detritus* larvae were recorded across a wide salinity gradient mainly in coastal sebkhas and salt marshes (13–66.2 g/L NaCl), with pH ranging from 7.10 to 8.55 and a temperature from 25 to 32 °C, confirming their strong halophily and tolerance to highly mineralised and organically enriched waters. *Aedes detritus* is a Palearctic species that is also commonly found in northern Europe, particularly in mesohaline coastal habitats [85]. Its southern range spreads out in North Africa where it is found in various saline environments [16].

It is suggested as a vector of myxomatosis virus [62] and shown to transmit chikungunya virus [154], Rift Valley fever virus [87] and West Nile virus [10] under laboratory conditions.

###### *Aedes* (*Ochlerotatus*) *dorsalis* (Meigen, 1830)

*Aedes dorsalis* was recorded in northern and eastern Tunisia in 2019 (Nabeul, Bizerte, Sousse, Tunis, Ben Arous). Only a few larvae were detected, mainly in coastal and peri-urban saline/brackish habitats (sebkha margins and shallow pools <0.5 m), with salinity ranging from 0.1 to 11.3 g/L NaCl and near-neutral pH (7.0–7.3). These sites were associated with halophytic vegetation (*e.g. Sarcocornia fruticosa*, *Juncus maritimus*) and larvae were sometimes found alongside *Ae. caspius* and *Ae. detritus.* Morphologically it is challenging to distinguish *Aedes dorsalis* from *Ae*. *caspius* in the larval stage, but more conclusive at the adult stage. *Aedes dorsalis* is present throughout northern Europe and has also been reported in Morocco [16] and Egypt [116].

###### *Aedes* (*Dahliana*) *echinus* (Edwards, 1920) and *Aedes* (*Dahliana*) *geniculatus* (Olivier, 1791)

*Aedes echinus* and *Ae. geniculatus* are both phytotelmic species found mainly in tree holes in the cork oak forests of northwestern Tunisia [36°47′ N; 8°40′ E]. *Aedes geniculatus* larvae have also been collected in some containers holding organic and tannin-rich water [37°12′39.20″ N; 9°33′32.98″ E]. Both species are considered to be rare in Tunisia, where they are usually restricted to forested areas with sufficient tree cover. These species have a wide Palearctic distribution from Norway to Morocco and from Portugal to Kazakhstan [33].

###### *Aedes* (*Ochlerotatus*) *mariae* (Sergent & Sergent, 1903)

This species was found in rock pools on the rocky northern Tunisian coast where it emerges after seawater floods rock pools. Immatures develop in these salty rock holes (we observed a salinity of 39 g/L NaCl), filled by sea spray [37°17′59.33″ N; 9°33′48.25″ E]. These sites are generally enriched with organic matter (dead algae, phytoplankton, various organic debris, *etc.*). The larvae can often tolerate wide variations in temperature. While it remains relatively rare, *Aedes mariae* is nonetheless found around the western Mediterranean basin, especially from Morocco [5, 151] to its northeastern limit in Tunisia [84].

###### *Aedes* (*Ochlerotatus*) *pulcritarsis* (Rondani, 1872)

Larvae of *Aedes pulcritarsis* were collected in both humid [37°20′10.19″ N; 9°40′6.99″ E] and subhumid zones [37°4′2.95″ N; 11°1′35.75″ E]. This rare species breeds primarily in the tree holes of the cork oak forests of northern Tunisia. It is frequently found in association with other arboreal species, such as *Orthopodomyia pulcripalpis*, *Ae*. *echinus*, *Ae*. *berlandi* and *Ae*. *geniculatus*. *Aedes pulcritarsis*, originally described from specimens collected in Italy, is distributed throughout the Palearctic region and has also been reported in Algeria [119] and Morocco [48]. The species is not known to play a role in disease transmission and therefore poses no public health threat [90].

###### *Aedes* (*Aedimorphus*) *vexans* (Meigen, 1830)

During our field investigations, the multivoltine mosquito species, *Aedes vexans*, was mainly observed along the banks of richly vegetated wadis and shallow temporary pools that form after seasonal rains. These habitats are located in central Tunisia (Sousse and Kairouan governorates), and provide a favourable local environment for this species (Fig. S6). *Aedes vexans* has been reported in Morocco [48] and Libya [124]; its range extends to the Palaearctic, Nearctic, Eastern and Australian regions [34]. The development cycle of *Ae*. *vexans* is rapid, and adults are capable of long-distance travel, making them a notable nuisance. During our study, this species was mainly found in springs. The females can live up to two months and feed on a wide range of hosts, biting humans, livestock, and birds throughout the day, especially at sunset. *Aedes vexans* is of medical interest because it has demonstrated laboratory vector competence for West Nile virus and its long-standing association with Tahyna virus and Rift Valley Fever virus circulation, supporting its potential role as a bridge vector where it occurs [34, 91, 170],

###### *Aedes* (*Fredwardsius*) *vittatus* (Bigot, 1861)

During our survey, *Aedes vittatus* was recorded in central Tunisia, particularly in the wadis of the arid Sidi Bouzid and the oasis streams of Tozeur. In contrast, a single earlier record documented this species in northeastern Tunisia (Kelibia) [155] (Fig. S6). Our result suggests that the range for *Ae. vittatus* in Tunisia could be broader, extending from the semi-arid central regions to oasis habitats in the south. In North Africa, *Ae. vittatus* has been collected from Morocco [48]. It is present in the Mediterranean basin, the Ethiopian region and the eastern region [112, 142]. This species is a competent vector for several viruses, including chikungunya virus, dengue virus, West Nile virus, yellow fever virus, and Zika virus. Its ability to transmit these viruses has been demonstrated primarily in laboratory settings [35, 89, 91, 92, 141].

##### Genus *Culex* Linnaeus*,* 1758 [Tribe *Culicini* Meigen*,* 1818]

In the present study, we identified 9 of the 12 documented species of the genus *Culex* found in Tunisia: *Cx. deserticola*, *Cx. hortensis*, *Cx. impudicus*, *Cx. laticinctus*, *Cx. mimeticus*, *Cx. perexiguus*, *Cx. pipiens*, *Cx. pusillus* and *Cx. theileri*. All of these species have been recorded in neighbouring countries Algeria [82] and Morocco [151]. Of the species cited in much earlier literature, *Cx. antennatus*, *Cx. territans* and *Cx. univittatus* were not found during our surveys.

###### *Culex* (*Maillotia*) *deserticola* Kirkpatrick, 1925

**Figure S9.**
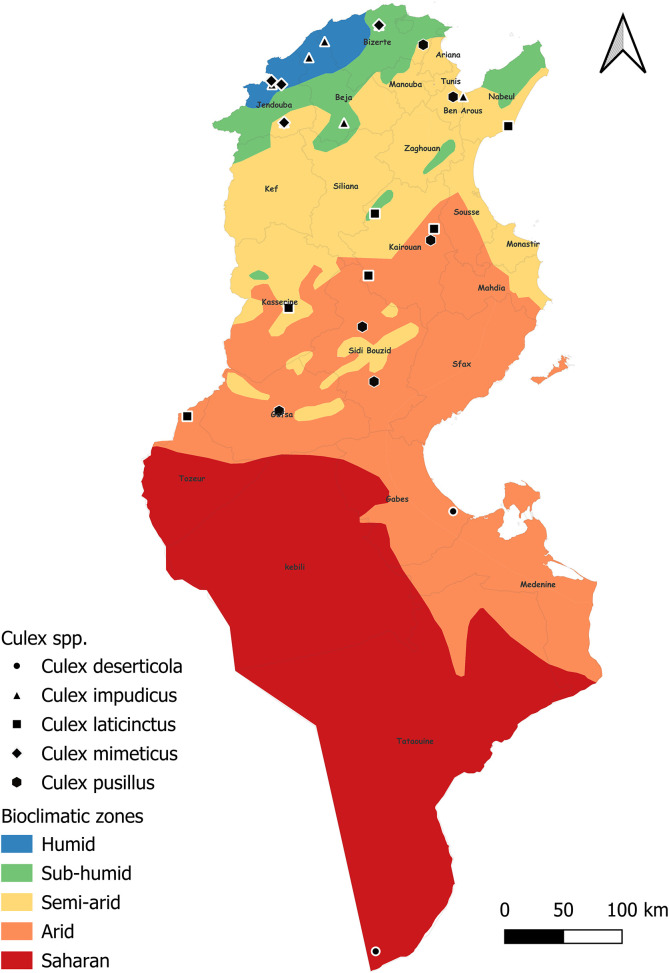
Geographical distribution of different *Culex* species [*Culex deserticola*, *Culex impudicus*, *Culex laticinctus*, *Culex mimeticus* and *Culex pusillus*] in Tunisia (2013–2023) across the different bioclimatic zones.

*Culex deserticola* was collected predominantly in central Tunisia, particularly in pre-Saharan regions (Fig. S9). *Culex deserticola* is highly adapted to arid environments and typically breeds in wadi beds and pools with sandy bottoms, where the water is fresh and clear and vegetation is limited. The larvae are found most commonly during the winter and spring seasons. The species continues to be considered rare overall with a distribution that extends from northern Africa to Iran [56].

###### *Culex* (*Maillotia*) *hortensis* Ficalbi, 1889

During our investigation, we collected *Cx. hortensis* in the north during the spring and autumn, seasons during which the species is active. Larvae were found only once in a habitat [36°46′25.03″ N; 8°41′5.09″ E] with cool, fresh water and without significant vegetation cover. *Culex hortensis* is considered to be rare in Tunisia, with populations restricted mainly to higher elevations and cooler environments. The species is widely distributed throughout the North African countries, including Algeria [124] and Morocco [48]. As females predominantly feed on amphibians and reptiles, the species has no significant public health implications [109].

###### *Culex* (*Neoculex*) *impudicus* Ficalbi, 1890

During our surveys, we collected *Cx. impudicus* in humid and sub-humid regions of Tunisia where they were found in small, shaded ponds with cool, clear fresh water (Fig. S9). It has distinct seasonal activity; its larvae are typically found from March to October*. Culex impudicus* is considered to be common in northern Tunisia where most populations are concentrated in forested areas, where suitable habitats remain relatively undisturbed. This mosquito is known for being batracophilic, feeding mainly on amphibians and other cold-blooded vertebrates, and poses no significant public health risk to humans [6]. In North Africa, it has been documented in Algeria and Morocco [77, 88]. It is also widely distributed across the Mediterranean region, extending as far east as Iran [108].

###### *Culex* (*Culex*) *laticinctus* Edwards, 1913

During our investigations, *Cx. laticinctus* was collected mainly in Tunisia’s arid and Saharan regions **(**Fig. S9). We also detected the species further north (*e.g.* near Hammamet), suggesting a broader distribution than previously reported. It was found at high densities along wadi banks and streams with slightly saline water. Where physicochemical data were available, *Cx. laticinctus* was detected in low-salinity inland habitats (≈2.0–2.8 g/L NaCl; pH ~7.6 where measured), such as wadi-bed depressions. Although previously considered to be relatively common, particularly in arid regions, our data suggest more restricted current distribution. This species is not considered a primary vector of human pathogens. The distribution of *Cx. laticinctus*, primarily in the Mediterranean region, ranges from the Canary Islands to the Middle East [105].

###### *Culex* (*Culex*) *mimeticus* Noè, 1899

Our findings show that *Cx*. *mimeticus* is present in localities in northern Tunisia (Fig. S9). It was previously reported as being restricted to the northwest [18]. Its distribution is more closely associated with lowland areas rather than specific bioclimatic zones. The larvae develop in wadis and shallow temporary pools where the water is generally fresh and cool, and commonly colonised by filamentous algae. They are usually found in low numbers, making this species rare in Tunisia. *Culex mimeticus* is a montaneous species in the Mediterranean subregion and eastern region [139]. It has also been reported in North Africa, including Algeria and Morocco [56, 82, 151]. Adult females may seek refuge in houses, but bite neither humans nor other mammals.

###### *Culex* (*Culex*) *perexiguus* Theobald, 1903

**Figure S10.**
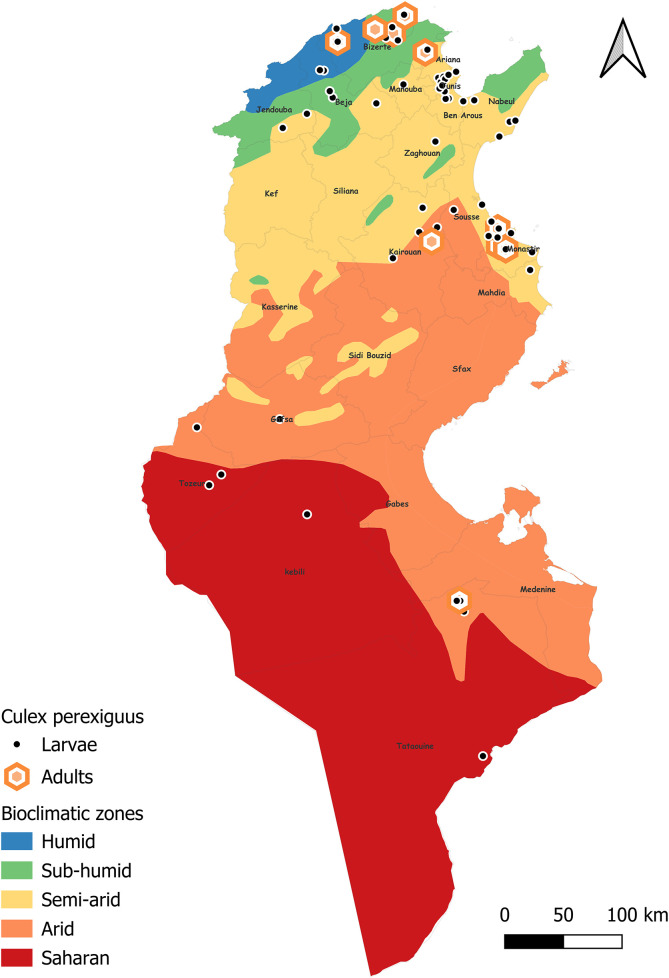
Geographical distribution of *Culex perexiguus* in Tunisia (2013–2023), by life stage (larvae and adults) across the different bioclimatic zones.

*Culex perexiguus* was one of the key species identified during our survey, with collections spanning all bioclimatic zones of Tunisia (Fig. S10). This primarily ornithophilic species breeds in both natural and artificial habitats. While it feeds mostly on birds, records exist of it entering homes and biting humans at night. In habitats where physico-chemical measurements were taken, *Cx. perexiguus* was primarily associated with waters having salinity ranging from 0.5 to 2.6 g/L NaCl and pH values of 7.08–8.9, including sites fed by springs and canals connected to sebkhas.

The species is considered to be common in Tunisia, where we found it to coexist with *Cx. pipiens* during the summer and autumn months. *Culex perexiguus* is an important vector of arboviruses, including West Nile virus and Sindbis virus, both of which have been detected in this species in Tunisia [83]. Its role in the transmission of these viruses has been established in other North African countries, including Egypt [46]. First described in 1903, *Cx*. *perexiguus* has been confused with *Cx*. *decens*, *Cx*. *pallidocephalus* and especially with *Cx*. *univittatus* [55]. Harbach (1988) proposed criteria to clearly distinguish these different taxa. The distribution of *Cx*. *perexiguus* extends from Morocco to India [3] and it has been recorded in all countries of North Africa [17].

###### *Culex* (*Culex*) *pipiens* Linnaeus, 1758

**Figure S11.**
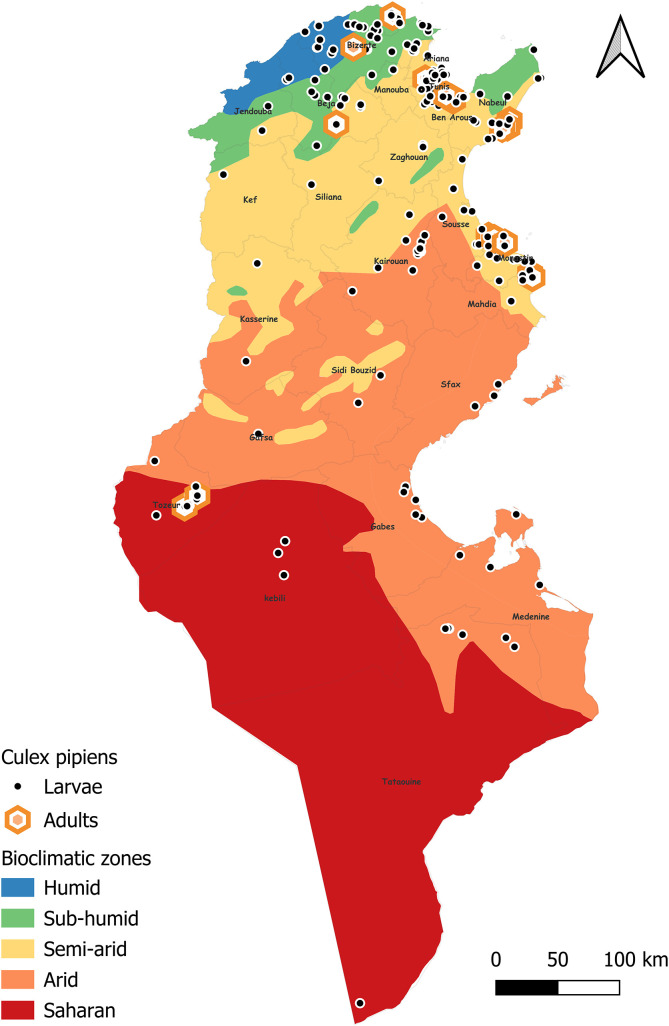
Geographical distribution of *Culex pipiens* in Tunisia (2013–2023), by life stage (larvae and adults) across the different bioclimatic zones.

This species is widespread and abundant in Tunisia. It was collected in all bioclimatic zones from various water bodies, with populations observed in both urban and rural areas. Its ecological plasticity explains its adaptability to breeding in various environments under different environmental conditions, making it one of the most widespread mosquito species in the country (Fig. S11). Importantly, *Cx*. *pipiens* larvae have been found in a variety of natural habitats, including wadi banks, swamps, shallow temporary pools, hill lakes, ponds, irrigation channels and streams. It also breeds readily in man-made water areas such as canals, pools, unused wells, ditches, catch basins, unused tyres, open-air wastewater and rainwater canals, ditches, water troughs, crawlspaces and drainage canals. In the subset of characterised larval habitats, *Cx. pipiens* was recorded mainly in low-salinity inland wadis (0.5–2.6 g/L NaCl; pH 7.08–7.7) but also occurred in brackish coastal sebkha/lagoon environments (up to 28–32 g/L NaCl; pH ~7.47–8.5), supporting its broad ecological plasticity. Its distribution Maghreb-wide is well documented, with records in all countries [17, 18, 108, 151]. Globally, *Cx. pipiens* is widely distributed across temperate and tropical regions, including Europe, Africa, Asia and North America. *Culex pipiens* is a well-known vector of West Nile virus and thus particularly important from a public health perspective. This species was implicated in the 1997 and 2003 outbreaks of West Nile fever in Tunisia, during which 20 deaths were reported [47, 153]. Additionally, *Cx. pipiens* is a potential vector of other arboviruses, including Rift Valley fever virus, especially during periods of increased rainfall and standing water [40, 127].

###### *Culex* (*Barraudius*) *pusillus* Macquart, 1850

Our findings indicate that *Cx. pusillus* is a halophilic species, found only in saline, vegetation-rich environments, where its larvae develop. This species was previously considered to be rare in Tunisia and recorded only in the south, but it is now also present in the centre of the country, suggesting a shift in its distribution (Fig. S9). This change is likely driven by habitat changes resulting from increased drought, which may be creating new ecological niches northward.

In North Africa, it has also been documented in Algeria [118] and Egypt [1], and extends to the southern-most Palearctic region [108].

During the course of our research, we noted that females of this species were not aggressive towards humans. Of note, *Cx. pusillus* is not known to bite humans and is thus not considered a significant public health threat.

###### *Culex* (*Culex*) *theileri* Theobald, 1903

**Figure S12.**
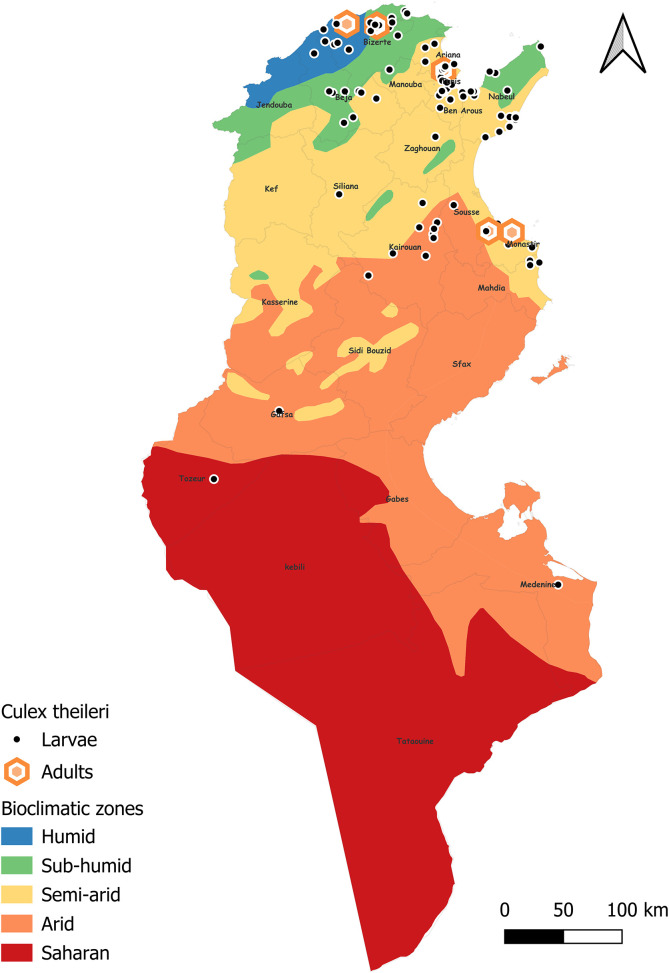
Geographical distribution of *Culex theileri* in Tunisia (2013–2023), by life stage (larvae and adults) across the different bioclimatic zones.

*Culex theileri* is another mosquito species that we identified in all bioclimatic zones during this investigation, particularly in a wide range of breeding habitats (Fig. S12). Like *Cx. pipiens*, *Cx*. *theileri* displays great ecological flexibility, inhabiting both natural and artificial bodies of water (wadi banks, swamps, shallow pools, ponds, streams and irrigation channels). It can tolerate varying levels of salinity, even breeding in brackish water, which expands its distribution across arid and semi-arid regions of the country. *Culex theileri* is considered to be common in Tunisia and has a broad geographic distribution range; we observed significant populations across rural and agricultural areas*.* In the characterised sites, *Cx. theileri* occurred from low-salinity inland habitats (0.5–2.6 g/L NaCl; pH 7.08–7.7) to more mineralised/brackish waters (*e.g.*, 4.3 g/L NaCl, pH 8.8; and up to 25 g/L NaCl, pH 8.5), consistent with its tolerance to salinity gradients. Historically, *Cx. theileri* was noted for its wide distribution in North Africa, with reports in Algeria [136], Morocco [151] and Libya [66]. Globally, it is found from southern Europe to the Middle East, and as far east as Afghanistan. The distribution of *Cx. theileri* has remained relatively stable, although it has been found more frequently in semi-arid regions where brackish water habitats have been expanded by agricultural practices. Although this species has been recorded as a potential vector of West Nile virus, its public health importance in Tunisia relative to other vector species like *Cx. pipiens* remains unclear [162].

##### Genus *Culiseta* Felt, 1904 [Tribe Culisetini Belkin, 1962]

Among the five species of the genus *Culiseta* (*Cs*.) mentioned in Tunisia, we collected only two during our survey, namely *Cs. longiareolata* and *Cs. subochrea*. Brunhes *et al*. (2000) [17] mentioned the presence of *Cs. annulata* and *Cs. fumipennis* in Tunisia, and Robert *et al*. (2019) [108] listed *Cs. morsitans* in the country. However, we found none of these species during our investigations.

###### *Culiseta* (*Allotheobaldia*) *longiareolata* (Macquart, 1838)

**Figure S13.**
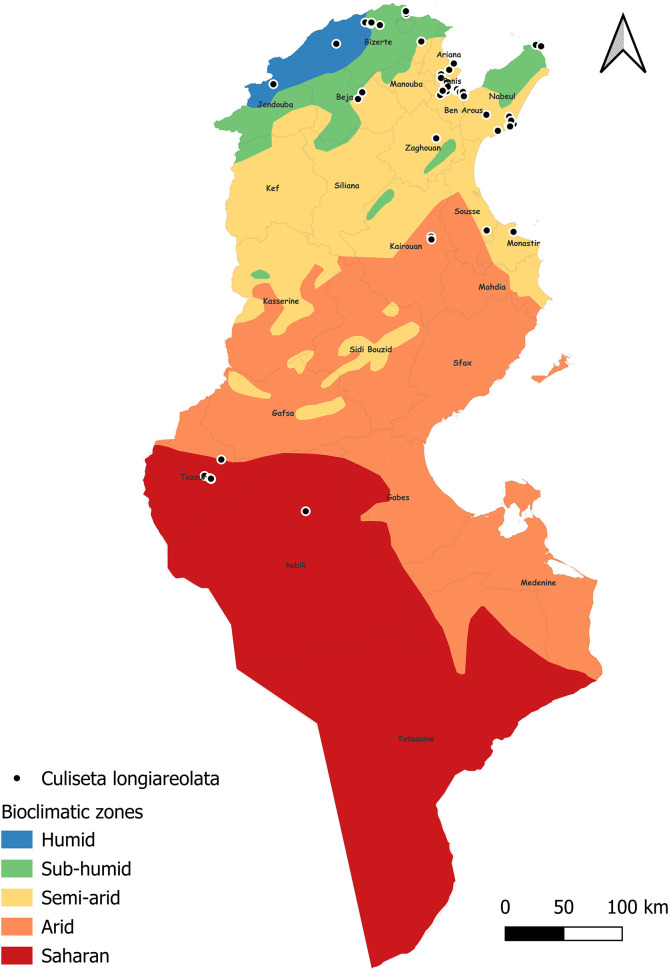
Geographical distribution of *Culiseta longiareolata* in Tunisia (2013–2023) across the different bioclimatic zones.

During our surveys, the larvae of *Cs. longiareolata* were collected in a variety of habitats spanning both natural breeding sites like wadi banks and swamps and artificial sites such as canals, unused wells, catch basins, discarded tyres and irrigation ditches. In the subset of characterised habitats, *Cs. longiareolata* was also detected in brackish sites (*e.g.*, 32 g/L NaCl; pH 8.5), co-occurring with *Cx. pipiens* and *Ae. detritus*. This adaptability to diverse breeding environments contributes to the widespread distribution and notable abundance of *Cs. longiareolata* across all bioclimatic zones in Tunisia (Fig. S13). Widely distributed throughout the southern Palearctic, as well as the Eastern and Afro-tropical regions, *Cs. longiareolata* has a particularly high prevalence in North Africa [18]. In Tunisia, it often shares habitats with *Cx. pipiens* [20, 105, 119, 137, 158, 159]. Compared with previous surveys, our study suggests an expanded range and confirms the species’ abundance across various Tunisian habitats where it feeds primarily on birds, serving as a vector for avian blood parasites. It rarely targets humans [6].

###### *Culiseta* (*Culiseta*) *subochrea* (Edwards, 1921)

The larvae of *Cs. subochrea* develop in fresh water pools with low organic matter; however, they can also tolerate brackish water, as they are found in irrigation ditches lined with *Salicornia*, in association with *Ae. detritus* and *Ae. caspius*. During our investigations, we identified *Culiseta subochrea* exclusively in the oases of Tozeur [33°54′54.71″ N; 8°7′49.38″ E]. Although currently rare, this species was once more broadly distributed according to historical records [18]. *Culiseta subochrea* has been reported in North Africa, except in Libya, and penetrates deep into desert regions [88]. It is a widely distributed species found throughout Europe, but appears to be more abundant in the southern Palearctic region [93].

##### Genus *Orthopodomyia* Theobald, 1904 [Tribe Orthopodomyiini Belkin, Heinemann & Page, 1970]

###### *Orthopodomyia pulcripalpis* (Rondani, 1872)

**Figure S14.**
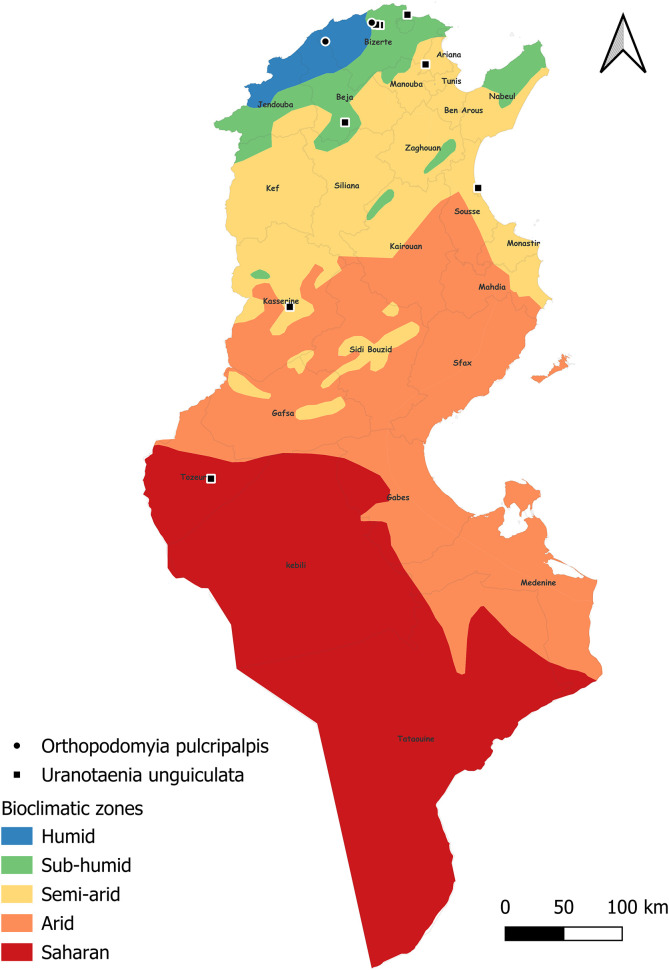
Geographical distribution of *Orthopodomyia pulcripalp*is and *Uranotaenia unguiculata* in Tunisia (2013–2023) across the different bioclimatic zones.

*Orthopodomyia pulcripalpis* is a rare arboreal species found in northern forests in humid and sub-humid zones (Fig. S14). We found larvae in cork oak tree holes. This species is present throughout Western Europe and the Mediterranean Palearctic sub-region [6].

##### Genus *Uranotaenia* Lynch Arribálzaga, 1891 [Tribe Uranotaeniini Lahille, 1904]

###### *Uranotaenia* (*Pseudoficalbia*) *unguiculata* Edwards, 1913

*Uranotaenia unguiculata* larvae were observed from sub-humid to sub-Saharan zones (Fig. S14). The larvae breed primarily in shallow temporary pools with abundant upright vegetation such as poorly maintained canals, ponds and irrigation ditches. The water in these pools is fresh to slightly salty. Adults are frequently collected in early autumn. Females of *Ur. unguiculata* transmit parasites to amphibians and reptiles. In Tunisia, several authors have reported this common species [124, 156]. *Uranotaenia unguiculata* has been reported in North Africa from Morocco to Egypt [58]. In addition to the Mediterranean region, it is also found in Central Europe and the Middle East [111].

#### Species documented in the literature but not collected in our investigations (see Supplementary Tables 1–3 for details)

##### *Anopheles* (*Anopheles*) *coustani* Laveran, 1900

Despite historical citations of its existence in Tunisia [20, 132], *An. coustani* was not recorded in our investigations. *Anopheles coustani* belongs to the *Coustani* group, which includes morphologically similar species such as *An.* (*Ano.*) *ziemanni* Grünberg, 1902. Robert *et al*. (2019) [108] included *An. coustani* in their regional checklist based on outdated data, without recent confirmation. These records are now considered to be questionable. Several studies suggest that earlier identifications of *An. coustani* in North Africa, including Tunisia, may have resulted from misidentifications with *An. ziemanni*, a species with overlapping morphological characteristics [151].

##### *Anopheles* (*Cellia*) *dthali* Patton, 1905

*Anopheles dthali* has been historically reported in the Maghreb, particularly in the southern regions of Tunisia and Morocco [124]. This species prefers desert or semi-desert environments, and typically breeds in temporary water bodies [54]. In Tunisia, *An. dthali* has been recorded mainly in the southern part of the country, and is considered rare, with previous records from arid zones [14, 36]. *Anopheles dthali* was not detected during our surveys. This may reflect a decline in local populations linked to prolonged drought and habitat loss, but its persistence at low densities or in unsampled microhabitats cannot be ruled out. Although not considered a primary vector, *An. dthali* has been implicated in the transmission of *Plasmodium* in limited reports from North Africa, and its vectorial capacity remains poorly documented [23].

##### *Anopheles* (*Cellia*) *superpictus* Grassi, 1899

*Anopheles superpictus* was reported in eastern Tunisia, particularly on the banks of wadis [63, 161], but we did not detect it during our investigations. This apparent absence may reflect a decline in local populations, due probably to environmental changes that may have severely limited species-suitable breeding sites. Larvae usually develop in river bed pools [112]. *Anopheles superpictus* is widely distributed across the northern Mediterranean region, from North Africa to the Middle East [108], where it is well known as a malaria vector [138].

##### *Aedes* (*Stegomyia*) *aegypti* (Linnaeus, 1762)

*Ae. aegypti* was reported in Tunisia in several cities [20, 114, 166], but it disappeared in the early 1960s. Despite its absence in our surveys, this species must remain under surveillance because it can thrive in urban environments and plays a role as a vector of several arboviruses such as chikungunya, dengue, yellow fever and Zika viruses [44, 95, 147].

##### *Aedes* (*Ochlerotatus*) *albineus* Séguy, 1923

*Aedes albineus* was first described by Séguy (1923) [115] from adult specimens collected in southern Algeria. However, its taxonomic status remains uncertain due to the loss of type material and limited original descriptions. While it resembles *Ae. caspius*, this species is paler and lacks the hook-shaped setae on the male coxite. Some authors (*e.g.*, Knight and Stone, 1977) [69] considered it a synonym of *Ae. caspius*, whereas others [17, 74] suggested it may correspond to pale forms of *Ae. caspius* observed in desert regions. Its absence in our current surveys could be due to habitat changes or past misidentifications. Further molecular studies are needed to clarify its status and potential occurrence in Tunisia.

##### *Aedes* (*Aedes*) *cinereus* Meigen, 1818

Robert *et al*. reported (2019) [108] *Aedes cinereus* from Tunisia but several authoritative reviews, such as Moussiegt, 1983 [84] and Brunhes *et al*., 2000 [18], did not include this species in the Tunisian mosquito fauna. Callot (1938) [20] noted that *Ae. cinereus* larvae are very similar morphologically to those of *Ae. vexans*, which may lead to misidentification. The presence of *Ae. cinereus* in Tunisia therefore remains doubtful, as no detailed descriptions of its morphology or breeding habitat have been documented to date. Under favorable climatic conditions, we cannot exclude its future occurrence in the country.

##### *Aedes* (*Acartomyia*) *zammitii* (Theobald, 1903)

Among the three species composing the Mariae Complex, two are found in North Africa (*Ae. mariae* and *Ae. zammitii*) [29]. Although *Ae. zammitii* was previously reported in Tunisia [17], we did not find this species during our surveys. Morphologically, *Ae. zammitii* is very similar to *Ae. mariae*, and both species share the same habitat niches of rock pools along the seashore. *Ae. zammitii* is found primarily on the eastern coasts of Italy, Sicily and Malta [28], and it is possible that previous records of *Ae. zammitii* in Tunisia were misidentifications of *Ae. mariae*. Alternatively, *Ae. zammitii* may have lost its range due to climatic changes, urbanisation, or other environmental factors. The presence of this species and of *Ae. cinereus* has not been confirmed in Morocco or Algeria.

##### *Culex* (*Culex*) *antennatus* (Becker, 1903)

*Culex antennatus* is a species widely distributed throughout the Afrotropical region and as far south as Madagascar. It has been recorded in North Africa, including Tunisia, under its synonym *Culex laurenti* Newstead, 1907 [55]. *Culex antennatus* has been documented in neighbouring countries such as Algeria and Morocco [17], but was not found during our surveys. This could reflect habitat degradation, particularly in freshwater sources impacted by drying and increased salinity, a consequence of climate change. This species may also have become rarer in northern Africa due to competition from other mosquito species better adapted to altered environments.

##### *Culex* (*Culex*) *territans* Walker, 1856

*Culex territans* has been documented in North Africa, especially in Tunisia. It usually breeds in small, clear ponds and has a marked preference for cooler, shaded habitats [18]. It often shares the same habitat as *Culex impudicus*. The absence of this species in our study could be indicative of the evolution of these specific microhabitats, which have been affected by habitat loss and urban development. One hypothesis is that environmental changes, such as the drying up of small ponds or the reduction in the availability of suitable shaded breeding sites have contributed to the apparent absence of the species [41].

##### *Culex* (*Culex*) *univittatus* Theobald, 1901

*Culex univittatus* was first reported and described in Tunisia by Callot (1938) [20], but its current presence remains uncertain. Robert *et al*. (2019) [108] suggested that earlier reports of *Cx. univittatus* in Tunisia may have resulted from misidentifications of *Cx. perexiguus*, due to their close morphological similarities. We found no *Cx. univittatus* in our current surveys, which could support the hypothesis of past misidentifications.

##### *Culiseta* (*Culiseta*) *annulata* (Schrank, 1776)

*Culiseta annulata* is a cold-adapted species with a broad distribution across Europe, parts of Asia, and North Africa [108]. In Tunisia, it was reported by Husson (1907) [60] and later by Colas-Belcour (1931) [27] in the oasis of Tozeur, and also noted in nearby regions such as Biskra in Algeria [20]. This species is easily recognisable by its wing spots and white leg bands. We found no *Culiseta annulata* in our investigations, possibly due to ecological changes such as desertification and salinisation affecting freshwater habitats, which may contribute to a shift in the composition of the mosquito community in Tunisia’s semi-arid zones.

##### *Culiseta* (*Culicella*) *fumipennis* (Stephens, 1825)

*Culiseta fumipennis* is a Euro-Mediterranean mosquito historically recorded in the humid and forested areas of northwestern Tunisia, notably in Ghardimaou and Aïn Draham, based on larval identifications and determinations by Senevet and Prunnelle (1928) [125]. It has also been reported from Algeria [52] and Morocco [151], confirming its presence in the humid bioclimatic zones of North Africa. Despite these earlier records, *Cs. fumipennis* was not detected during our investigations. This may reflect the degradation of its preferred habitats.

##### *Culiseta* (*Culicella*) *morsitans* (Theobald, 1901)

*Culiseta morsitans* has also been reported to be present in Tunisian mosquito fauna [17, 108]. In reality, Brunhes *et al*. (2000b) [17] had already excluded this species from the list of mosquitoes of the Maghreb. Similarly, Moussiegt (1983) [84] made no mention of *Cs. morsitans* in Tunisia. Historically, Senevet and Prunnelle (1928) [125] reported having examined 18 larvae of *Cs. morsitans* collected by Gauthier in a marsh located in the former Lake Halloula (Algeria). In Libya, Ghidini (1934) [49] found larvae of this species in Oued Tmini. Later, Gaud (1953) [48] and Trari (1991) [151] noted that they had never encountered this species in Morocco, despite apparently favourable climatic conditions in the country. Taken together, these findings suggest that the presence of *Culiseta morsitans* in Tunisia remains highly doubtful.

## Conclusion

During our investigations, we observed 35 mosquito species belonging to 6 genera, of the 49 reported in the literature. It is certain that some species, such as *Ae. aegypti* have disappeared completely, while others such as *An. dthali* and *Ae. albineus* have become rare, especially since Tunisia has experienced record water shortages and extremely hot summers over the past decade as a result of climate change. The expanding human population has resulted in the construction of buildings and infrastructure and the introduction of roads, which have had a detrimental effect on the natural environment. The improper disposal of wastewater, often discharged into waterways without any form of treatment, exacerbates the situation. These environmental changes have a significant impact on the mosquito habitats whose disruption is a key factor in the observed changes in population diversity; the disappearance of many ponds, marshes and lakes, and the drying up of wadis and certain water sources. Consequently, several larval breeding sites have disappeared. We found that some stagnant water points have become very polluted and are suitable only for *Cx. pipiens*.

Our work provides an overview of the mosquito species present in Tunisia, their current distribution and their favourable habitats, thus facilitating investigations should arboviroses circulate. From a taxonomic and nomenclatural point of view, it remains important to update classifications and identifications. The implementation of new identification techniques using genetic/molecular approaches and the use of new techniques such as matrix-assisted laser desorption/ionisation time-of-flight MALDI-ToF mass spectrometry will allow for rapid and accurate identification of specimens and potentially highlight cryptic species, such as those of the Detritus or Mariae complexes or others with a similar morphology that create identification problems. It is also notable that compared to previous records, the number of species has decreased, potentially due to misidentifications such as *An. coustani*, *An. ziemanni* and *Cx. univittatus*. Additionally, some species have changed their distribution areas, such as *An. petragnani*, which has recently been found in central Tunisia, and *Ae. mariae*, which now occupies more habitats along the northern coasts. Finally, monitoring the spread of the recently introduced and invasive *Ae. albopictus* is necessary given its role as a vector for several arboviruses. The introduction of other species such as *An. stephensi* should also be surveyed.

## Data Availability

The data used to support the findings of this study are available from the corresponding author upon request.

## Supplementary material

*Table S1.* Natural and artificial breeding sites of mosquito species in Tunisia across bioclimatic zones. *Table S2*. Checklist of mosquito species (Diptera: Culicidae) in Tunisia based on field collections (2013–2023) and literature records. *Table S3*. Historical and bibliographic records of mosquito species in Tunisia. Table S3 includes many references, including [15, 21, 22, 30, 31, 39, 51, 71, 75, 76, 94, 102, 103, 107, 117, 121, 126, 128, 129, 130, 131, 133, 134, 135, 148, 157, 160].

The supplementary material of this article is available at https://www.parasite-journal.org/10.1051/parasite/2026018/olm.
