# Supplementary material for: The mosquitoes (Diptera: Culicidae) of Tunisia: updated checklist and new distribution data
Source: Parasite. 2026 Apr 14;33:20. doi: 10.1051/parasite/2026018 (PMC13078122; doi:10.1051/parasite/2026018)
Supplement: Supplementary file 1 — Table S1. Natural and artificial breeding sites of mosquito species in Tunisia across bioclimatic zones. [file parasite-33-20-s1.pdf]

Supplementary Table 1. Natural and artificial breeding sites of mosquito species in Tunisia across bioclimatic zones

Distribution zone	Mosquito species	Main natural breeding site(s)	Main artificial breeding site(s)
**All bioclimatic**	*Aedes caspius*	Wadi bank (beds), marshes, swamps, shallow temporary pools, ponds, sabkhas (mudflat)	Hill lakes, ditches
	Aedes coluzzii	Sabkhas	
	Aedes detritus	Wadi bank (beds), marshes, swamps, shallow temporary pools, ponds, sabkhas	Hill lakes, ditches
	Aedes dorsalis	Sabkhas	Water puddle
	Anopheles algeriensis	Wadi bank (beds), swamps, shallow temporary pools, ponds, marshes, slow-moving streams	Hill lakes, ditches
	Anopheles cinereus	Wadi bank (beds), swamps, ponds, slow-flowing waters	Pools, ditches, catch basins
	Anopheles labranchiae	Wadi bank (beds), swamps, shallow temporary pools, ponds, freshwater or slightly salty, sunny habitats dominated by vegetation of the *Ranuncaulus* and *Cartophyllum*	Hill lakes, ditches, quarries
	Culex pipiens	Wadi bank (beds), Swamps, shallow temporary pools, ponds, sabkha, streams, sow-moving streams, irrigation channels	Hill lakes, unused tyres, drainage canals, quarries, crawlspaces, water troughs, open-air wastewater and rainwater canals, pools, unused wells, ditches, catch basins,
	Culex perexiguus	Wadi bank (beds), swamps, shallow temporary pools, ponds, large temporary or permanent marshes, sanitary empties (highly polluted water), tree cavities, drainage water, puddles in streams	Hill lakes, irrigation canals, water troughs
	Culex theileri	Wadi bank (beds), swamps, shallow temporary pools, ponds, streams, marshes rich in vegetation	Hill lakes, unused tyres, irrigation canals, quarries
	Culiseta longiareolata	Wadi bank (beds), swamps, shallow temporary pools, ponds	Hill lakes, unused tyres, quarries, livestock drinker, ditches, unused wells
	Culiseta subochrea	Wadi bank (beds), swamps, shallow temporary pools, ponds	Pools, ditches, water troughs,livestock drinker
	Uranotaenia unguiculata	Wadi bank (beds), swamps, shallow temporary pools, ponds,	Canals (open-air wastewater and rainwater), ditches

		shallow freshwater	
**Humid and subhumid**	*Aedes albopictus*	Streams, streams slow-moving	Pools, unused tyres, water vessels, artificial containers - small water tanks
	Aedes berlandi	Tree holes, tree litter (basic pH)	-
	Aedes echinus	Tree holes	-
	Aedes geniculatus	Tree holes, rock holes	Pools
	Aedes mariae	Rock holes (in coastal areas)	-
	Aedes pulcritarcis	Tree holes (oaks)	-
	Anopheles claviger	Ponds, streams, streams slow-moving, freshwater or slightly brackish water site	-
	Anopheles marteri	Streams, streams slow-moving, mountain breeding sites, the banks of waterfalls	-
	Anopheles petragnani	Streams, streams slow-moving	-
	Anopheles plumbeus	Tree holes	-
	Anopheles ziemmani	Wadi bank (beds), marshland, grassy edge of large ponds	-
	Culex hortensis	Wadi bank (beds), streams, shallow temporary pools	-
	Culex impudicus	Streams, shallow temporary pools, riverbanks, ponds, grassy ditches	Ditches
	Culex mimeticus	Wadi bank (beds), streams, shallow temporary pools, puddles that form in wadi beds	-
	Orthopodomyia pulcripalpis	Tree holes	-
**Arid and saharian**	*Aedes dorsalis*	Wadi bank (beds), swamps, ponds	Pools, ditches
	Aedes vexans	Wadi bank (beds), shallow temporary pools, flooded marshes, river inlets, flooded fields	-
	Aedes vittatus	Wadi bank (beds), streams	-
	Anopheles	Wadi bank (beds)	Canals, pools

	multicolor		
	Anopheles sergentii	Wadi bank (beds), streams, small permanent wetland	Irrigation canals
	Culex deserticola	Wadi bank (beds)	Pools
	Culex laticinctus	Wadi bank (beds), streams, urban environment, temporary puddles, irrigation channels, springs, wells	-
	Culex pusillus	Wadi bank (beds)	-
