# Supplementary material for: The mosquitoes (Diptera: Culicidae) of Tunisia: updated checklist and new distribution data
Source: Parasite. 2026 Apr 14;33:20. doi: 10.1051/parasite/2026018 (PMC13078122; doi:10.1051/parasite/2026018)
Supplement: Supplementary file 2 — Table S2. Checklist of mosquito species (Diptera: Culicidae) in Tunisia based on field collections (2013–2023) and literature records. [file parasite-33-20-s2.pdf]

Supplementary Table 2: Checklist of mosquito species (Diptera: Culicidae) of Tunisia based on field collections (2013–2023) and literature records

Genus	Species	Recently found	Previously cited	Occurrence status
Anopheles	*Anopheles* (*Anopheles*) *algeriensis* Theobald, 1903	*	*	native
	Anopheles (*Cellia*) *cinereus* Theobald, 1901	*	*	native
	Anopheles (*Anopheles*) *claviger* (Meigen, 1804)	*	*	native
	Anopheles (*Anopheles*) *coustani* (Laveran, 1900		*	uncertain
	Anopheles (*Cellia*) *dthali* Patton, 1905		*	native
	Anopheles (*Anopheles*) *labranchiae* Falleroni, 1926	*	*	native
	Anopheles (*Anopheles*) *marteri* Senevet & Prunnelle, 1927	*	*	native
	Anopheles (*Cellia*) *multicolor* Cambouliu, 1902	*	*	native
	Anopheles (*Anopheles*) *petragnani* del Vecchio, 1939	*	*	native
	Anopheles (*Anopheles*) *plumbeus* (Stephens, 1828)	*	*	native
	Anopheles (*Cellia*) *sergentii* (Theobald, 1907)	*	*	native
	Anopheles (*Cellia*) *superpictus* Grassi, 1899		*	native
	Anopheles (*Anopheles*) *ziemanni* Grünberg, 1902	*	*	native
Aedes	*Aedes (Stegomyia) aegypti* (Linnaeus, 1762)		*	extinct
	Aedes (Ochlerotatus) albineus Séguy, 1923		*	native
	Aedes (Stegomyia) albopictus (Skuse, 1894)	*		invasive
	Aedes (Ochlerotatus) berlandi Séguy, 1921	*	*	native
	Aedes (Ochlerotatus) caspius (Pallas, 1771)	*	*	native
	Aedes (Aedes) cinereus Meigen, 1818		*	native
	Aedes (Aedes) coluzzii Rioux, Guilvard & Pasteur, 1998	*	*	native
	Aedes (Aedes) detritus (Haliday, 1833)	*	*	native
	Aedes (Ochlerotatus) dorsalis (Meigen, 1830)	*	*	native
	Aedes (Dahliana) echinus (Edwards, 1920)	*	*	native
	Aedes (Dahliana) geniculatus (Olivier, 1791)	*	*	native
	Aedes (Acartomyia) mariae (Sergent & Sergent, 1903)	*	*	native
	Aedes (Ochlerotatus) pulcritarsis (Rondani, 1872)	*	*	native
	Aedes (Aedimorphus) vexans (Meigen, 1830)	*	*	native
	Aedes (Fredwardsius) vittatus (Bigot, 1861)	*	*	native
	Aedes (Acartomyia) zammitii (Theobald, 1903)		*	native
Culex	*Culex* (*Culex*) *antennatus* (Becker, 1903)		*	native
	Culex (*Maillotia*) *deserticola* Kirkpatrick, 1925	*	*	native

	Culex (*Maillotia*) *hortensis* Ficalbi, 1889	*	*	native
	Culex (*Neoculex*) *impudicus* Ficalbi, 1890	*	*	native
	Culex (*Culex*) *laticinctus* Edwards, 1913	*	*	native
	Culex (*Culex*) *mimeticus* Noè, 1899	*	*	native
	Culex (*Culex*) *perexiguus* Theobald, 1903	*	*	native
	Culex (*Culex*) *pipiens* Linnaeus, 1758	*	*	native
	Culex (*Barraudius*) *pusillus* Macquart, 1850	*	*	native
	Culex (*Culex*) *theileri* Theobald, 1903	*	*	native
	Culex (*Neoculex*) *territans* Walker, 1856		*	native
	Culex (*Culex*) *univittatus Theobald, 1901*		*	uncertain
Culiseta	*Culiseta (Culiseta) annulata* (Schrank, 1776)		*	native
	Culiseta (Culicella) fumipennis (Stephens, 1825)		*	native
	Culiseta (Allotheobaldia) longiareolata (Macquart, 1838)	*	*	native
	Culiseta (Culiseta) subochrea (Edwards, 1921)	*	*	native
	Culiseta (Culicella) morsitans (Theobald, 1901)		*	native
Orthopodomyia	*Orthopodomyia pulcripalpis* (Rondani, 1872)	*	*	native
Uranotaenia	*Uranotaenia (Pseudoficalbia) unguiculata* Edwards, 1913	*	*	native
