# Supplementary material for: The mosquitoes (Diptera: Culicidae) of Tunisia: updated checklist and new distribution data
Source: Parasite. 2026 Apr 14;33:20. doi: 10.1051/parasite/2026018 (PMC13078122; doi:10.1051/parasite/2026018)
Supplement: Supplementary file 3 — Table S3. Historical and bibliographic records of mosquito species in Tunisia. Table S3 includes many references, including [15, 21, 22, 30, 31, 39, 51, 71, 75, 76, 94, 102, 103, 107, 117, 121, 126, 128, 129, 130, 131, 133, 134, 135, 148, 157, 160]. [file parasite-33-20-s3.pdf]

Supplementary Table 3: Historical and bibliographic records of mosquito species in Tunisia

Species	Authors having documented the species in Tunisia	References numbers
***Anopheles* (*Anopheles*) *algeriensis* Theobald, 1903**	Theobald (1901); Sergent (1909); Edwards (1921); Séguy & Bouvier (1924); Wassilieff (1934); Villain et al. (1935); Callot (1938); Vermeil (1950); Juminer (1959); Tabbabi et al. (2015)	[20,39,63,116,126,144,148,159,161,163]
***Anopheles* (*Cellia*) *cinereus* Theobald, 1901**	Edwards (1921); Séguy & Bouvier (1924); Callot (1938); Senevet et al. (1956); Rioux & Juminer (1963)	[20,39,105,116,123]
***Anopheles* (*Anopheles*) *claviger* (Meigen, 1804)**	Sicart (1942); Senevet et al. (1956); Boucheté et al. (1991)	[15,123,131]
***Anopheles* (*Anopheles*) *coustani* Laveran, 1900**	Villain et al. (1935); Callot (1938)	[20,161]
***Anopheles* (*Cellia*) *dthali* Patton, 1905**	Doby & Vermeil (1952); Bouattour et al. (1993)	[14,36]
***Anopheles* (*Anopheles*) *labranchiae* Falleroni, 1926**	Séguy & Bouvier (1924); Villain et al. (1935); Callot (1938); Sicart (1949, 1951a); Guy et al. (1976); Chadli et al. (1986); Bouattour et al. (1990, 1993); Boucheté et al. (1991); Tabbabi et al. (2015)	[13,14,20,25,51,116,132,133,144,161]
***Anopheles* (*Anopheles*) *marteri* Senevet & Prunnelle, 1927**	Sicart (1941); Senevet et al. (1956); Juminer (1959)	[63,123,130]
***Anopheles* (*Cellia*) *multicolor* Cambouliu, 1902**	Langeron (1921); Séguy & Bouvier (1924); Colas-Belcour (1931); Villain (1932); Wassilieff (1934); Villain et al. (1935); Callot (1938); Sicart (1949); Doby & Vermeil (1952); Senevet et al. (1956); Juminer (1959); Rioux & Juminer (1963); Chadli et al. (1986); Boucheté et al. (1991); Tabbabi et al. (2015)	[15,20,25,27,36,63,76,105,116,123,132,144,160,161,163]
***Anopheles* (*Anopheles*) *petragnani* del Vecchio, 1939**	Coluzzi et al. (1965)	[31]
***Anopheles* (*Anopheles*) *plumbeus* (Stephens, 1828)**	Senevet et al. (1955); Rioux et al. (1964)	[106,122]
***Anopheles* (*Cellia*) *sergentii* (Theobald, 1907)**	Villain et al. (1935); Callot and Ristorcelli (1938); Sicart (1949); Senevet et al. (1956); Juniner (1959); Chadli et al (1986); Boucheté et al. (1991); Tabbabi et al. (2015)	[15,22,25,63,123,132,144,161]
***Anopheles* (*Cellia*) *superpictus* Grassi, 1899**	Langeron (1921); Villain et al. (1935); Callot (1938); Juminer (1959)	[20,63,76,161]
***Anopheles* (*Anopheles*) *ziemanni* Grünberg, 1902**	Callot (1938); Senevet et al. (1956); Junimer (1959); Ramsdale (1990); Khalil (1980)	[20,63,67,96,123]
***Aedes (Stegomyia) aegypti* (Linnaeus, 1762)**	Séguy & Bouvier (1924); Callot (1938)	[20,116]

***Aedes (Ochlerotatus) albineus* Séguy, 1923**	Séguy (1923); Lambert et al. (1990)	[74,115]
***Aedes (Stegomyia) albopictus* (Skuse, 1894)**	Bouattour et al. (2019)	[12]
***Aedes (Ochlerotatus) berlandi* Séguy, 1921**	Rioux et al. (1964); Ben Ayed et al. (2019)	[8,106]
***Aedes (Ochlerotatus) caspius* (Pallas, 1771)**	Colas-Belcour (1931); Callot (1938); Vermeil (1950); Senevet et al. (1959); Juminer et al. (1964); Senevet & Andarelli (1964); Rioux et al. (1975); Krida et al. (2012); Ben Ayed et al. (2019); M'ghirbi et al. (2023)	[8,20,27,64,70,83,102,121,124,159]
***Aedes (Aedes) cinereus* Meigen, 1818**	Callot (1938)	[20]
***Aedes (Aedes) coluzzii* Rioux, Guilvard & Pasteur, 1998**	Rioux et al. (1998); Ben Ayed et al. (2019)	[8,104]
***Aedes (Aedes) detritus* (Haliday, 1833)**	Callot (1938); Vermeil (1953b, 1954); Juminer et al. (1964); Rioux et al. (1975); Pasteur et al. (1978); Krida et al. (2012); Ben Ayed et al. (2019); M'ghirbi et al. (2023)	[8,20,64,70,83,94,102,157,158]
***Aedes (Ochlerotatus) dorsalis* (Meigen, 1830)**	Séguy & Bouvier (1924); Colas-Belcour (1931); Callot (1938); Vermeil (1950); Senevet & Andarelli (1964);	[20,27,116,121,159]
***Aedes (Dahliana) echinus* (Edwards, 1920)**	Sicart (1952); Ben Ayed et al. (2019)	[8,134]
***Aedes (Dahliana) geniculatus* (Olivier, 1791)**	Langeron (1916); Ben Ayed et al. (2019)	[8,75]
***Aedes (Acartomyia) mariae* (Sergent & Sergent, 1903)**	Vermeil (1954); Coluzzi et al. (1974); Rioux et al. (1975); Bailly-Choumara (1968); Ben Ayed et al. (2019)	[5,8,30,102,158]
***Aedes (Ochlerotatus) pulcritarsis* (Rondani, 1872)**	Vermeil (1950); Sicart (1952a); Ben Ayed et al. (2019)	[8,134,159]
***Aedes (Aedimorphus) vexans* (Meigen, 1830)**	Callot (1938); Vermeil (1950); Ben Ayed et al. (2019)	[8,20,159]
***Aedes (Fredwardsius) vittatus* (Bigot, 1861)**	Vermeil (1953a); Boucheté et al., 1991; Ben Ayed et al. (2019)	[8,15,155]
***Aedes (Acartomyia) zammitii* (Theobald, 1903)**	Sicart (1952b)	[135]
***Culex* (*Culex*) *antennatus* (Becker, 1903)**	Harbach (1988)	[55]
***Culex* (*Maillotia*) *deserticola* Kirkpatrick, 1925**	Langeron (1921); Callot (1947); Senevet et al. (1959); Rioux & Juminer (1963); Harbach et al. (1988); Seurat (1942); Sicart (1953) Sicart (1954); Vermeil (1950); Vermeil (1951)	[21,55,76,105,124,128]
***Culex* (*Maillotia*) *hortensis* Ficalbi, 1889**	Callot (1947); Senevet et al. (1959); Vermeil (1950); Sicart (1953)	[21,124,136,159]
***Culex* (*Neoculex*) *impudicus* Ficalbi, 1890**	Sicart (1940, 1953), Senevet et al. (1959)	[124,129,136]

***Culex* (*Culex*) *laticinctus* Edwards, 1913**	Langeron (1921); Séguy (1928); Colas-Belcour (1931); Callot (1938); Vermeil (1950); Sicart (1953); Rioux & Juminer (1963)	[20,27,76,105,114,136,159]
***Culex* (*Culex*) *mimeticus* Noè, 1899**	Sergent (1909); Senevet & Prunnelle (1928); Sicart (1953), Senevet et al. (1959)	[124–126,136]
***Culex* (*Culex*) *perexiguus* Theobald, 1903**	Edwards (1921); Séguy & Bouvier (1924); Vermeil (1950); Sicart (1953); M'ghirbi et al. (2023)	[39,83,116,136]
***Culex* (*Culex*) *pipiens* Linnaeus, 1758**	Sergent (1909); Senevet & Prunelle (1928); Colas-Belcour (1931); Wassilieff (1934); Callot (1938); Vermeil (1950, 1954); Sicart (1953); Senevet et al. (1959); Rioux & Juminer (1963); Juminer et al. (1964); Krida et al. (1998); Beji et al. (2017)	[7,20,27,64,71,105,124–126,136,158,159,163]
***Culex* (*Barraudius*) *pusillus* Macquart, 1850**	Séguy & Bouvier (1924); Callot (1938); Sicart (1953); Abdel-Malek (1960)	[1,20,116,136]
***Culex* (*Culex*) *theileri* Theobald, 1903**	Séguy & Bouvier (1924); Sicart (1953); Senevet & Andarelli (1959); Rioux et al. (1975); Wasfi et al. (2016)	[102,116,124,136,162]
***Culex* (*Neoculex*) *territans* Walker, 1856**	Séguy & Bouvier (1924); Senevet (1947)	[116,117]
Culex* (*Culex*) *univittatus Theobald, 1901	Séguy & Bouvier (1924); Callot (1938)	[20,116]
***Culiseta (Culiseta) annulata* (Schrank, 1776)**	Senevet and Prunelle (1928); Callot (1938)	[20,125]
***Culiseta (Culicella) fumipennis* (Stephens, 1825)**	Callot (1938); Vermeil (1950)	[20,159]
***Culiseta (Allotheobaldia) longiareolata* (Macquart, 1838)**	Callot (1938); Vermeil (1950, 1954); Senevet et al. (1959); Sicart and Sardou (1961); Rioux & Juminer (1963)	[20,105,124,137,158,159]
***Culiseta (Culiseta) subochrea* (Edwards, 1921)**	Senevet & Andarelli (1960); Juminer et al. (1964); Rioux et al. (1968, 1974)	[64,103,107,119]
***Culiseta (Culicella) morsitans* (Theobald, 1901)**	Senevet and Prunelle 1928	[125]
***Orthopodomyia pulcripalpis* (Rondani, 1872)**	Callot (1938); Vermeil (1950); Senevet (1947)	[20,117,159]
***Uranotaenia (Pseudoficalbia) unguiculata* Edwards, 1913**	Callot (1938); Vermeil, (1953c); Senevet et al. (1959)	[20,124,156]
